# Integratively Genomic Analysis Reveals the Prognostic and Immunological Characteristics of Pyroptosis and Ferroptosis in Pancreatic Cancer for Precision Immunotherapy

**DOI:** 10.3389/fcell.2022.826879

**Published:** 2022-02-15

**Authors:** Ting Yu, Huaicheng Tan, Chunhua Liu, Wen Nie, Yang Wang, Kexun Zhou, Huashan Shi

**Affiliations:** ^1^ Department of Biotherapy, Cancer Center, West China Hospital, Sichuan University, Chengdu, China; ^2^ Department of Pathology and Laboratory of Pathology, State Key Laboratory of Biotherapy, West China Hospital, West China School of Medicine, Sichuan University, Chengdu, China

**Keywords:** pyroptosis, ferroptosis, pancreatic cancer, P-F score, immunity

## Abstract

The non-apoptotic cell death processes including pyroptosis and ferroptosis have been implicated in the progression and therapeutic responses of pancreatic adenocarcinoma (PAAD). However, the extent to which pyroptosis and ferroptosis influence tumor biology remains ambiguous, especially in PAAD, which is characterized with “cold” immunity. Considering the heterogeneity among different patients, it was more practical to quantify distinct cell death profiles in an individual tumor sample. Herein, we developed a pyroptosis-ferroptosis (P-F) score for PAAD patients in The Cancer Genome Atlas (TCGA) database. A high P-F score was associated with active immune phenotype, decreased genomic alterations, and significantly longer survival. Good accuracy of the P-F score in predicting overall survival (OS) was further confirmed in the TCGA-PAAD, ICGC-PACA-CA, and E-MTAB-6134 cohorts. Besides, one immunotherapy cohort (IMvigor210 dataset) has verified that patients with high P-F scores exhibited significant advantages in therapeutic responses and clinical benefits. The sensitivity to chemotherapeutics was analyzed through the Genomics of Drug Sensitivity in Cancer (GDSC), and patients with low P-F score might be more sensitive to paclitaxel and 5-fluorouracil. Collectively, the P-F score based on the systematic evaluation of cell death profiles could serve as an effective biomarker in predicting the outcomes and responses of PAAD patients to treatments with chemotherapeutic agents or immunotherapies.

## Introduction

As a lethal malignancy, pancreatic adenocarcinoma (PAAD) is characterized with a highly annual mortality rate that is close to the incidence rate (Gordon-Dseagu et al., 2018). Many challenges remain in the diagnosis and treatment of PAAD, with approximately 10% surviving 5 years after diagnosis (Siegel et al., 2020). During the past few decades, great progresses have been achieved in detection approaches and systemic treatment modality of PAAD, while only modest progress is made in patient prognosis. Even as the cornerstone of treatment for advanced PAAD, systemic chemotherapy still fails to control this disease (Sultana et al., 2007). Although brilliant clinical benefits have been observed with immunotherapy in multiple cancer types, PAAD that featured with “cold” immunity has proven to be insensitive to this approach (Brahmer et al., 2012; Neoptolemos et al., 2018). Therefore, classification of specific molecular subtypes of PAAD might provide assistance for precise and individual therapy to improve the outcomes of patients.

Current clinical challenges about PAAD mainly focus on the late diagnosis and resistance to the treatment-induced apoptosis (Tang et al., 2021). Therefore, targeting non-apoptotic cell death processes might develop promising strategies to conquer drug resistance and suppress tumor progression. Particularly, non-apoptotic cell death processes including pyroptosis and ferroptosis have been recently implicated in the progression and therapeutic responses of PAAD. As a lytic and inflammatory process, pyroptosis is a gasermins (GSDM)-mediated programmed necrosis, characterized with the activation of pro-inflammatory caspases and release of interleukin (IL) 1 family members (e.g., IL1β and IL18) (Cookson and Brennan, 2001). Some studies have reported the protumorigenic role of pyroptosis. Inflammatory mediators like IL1β released during the activation of pyroptosis might promote cancer stemness and progression (Vidal-Vanaclocha et al., 2000; Li et al., 2012). As key effectors of pyroptosis, gasdermin C and gasdermin D were found to be overexpressed in some cancers, which were associated with tumor progression and poor prognosis of patients (Miguchi et al., 2016; Gao et al., 2018). However, two recently published studies have shown the tumor-suppressive effect of pyroptosis through activating tumor immune microenvironment (TIME) (Wang et al., 2020; Zhang et al., 2020). Gasdermin E could facilitate the phagocytosis of tumor cells by macrophages and increase the number and efficiency of CD8^+^ T cells and natural killer (NK) cells, thereby inducing the pyroptosis of tumor cells and forming a positive feedback loop (Zhang et al., 2020). Moreover, ferroptosis, referred to as an iron-mediated accumulation of lipid peroxidation to lethal levels, was found to exhibit dual effects in the progression and suppression of PAAD (Tang et al., 2021). Immunotherapy-activated CD8^+^ T cells could suppress the tumor growth by enhancing ferroptosis-mediated lipid peroxidation in tumor cells (Wang et al., 2019). However, the ferroptosis induced by high-iron diets or Gpx4 (ferroptosis suppressor) depletion has been reported to activate the TMEM173/STING-dependent DNA sensor pathway and increase tumor-infiltrating macrophages, thereby promoting the *KRAS*-driven pancreatic tumorigenesis (Dai et al., 2020).

The crosstalk among pyroptosis, ferroptosis, and TIME is extremely complicated. Tumor cells undergoing pyroptosis could release gasdermin E and inflammatory factors to boost the infiltration of both tumor-suppressed immune cells such as CD8^+^ T cells and NK cells as well as tumor-promoting cells like myeloid-derived suppressor cells (MDSCs) (Zhang et al., 2020; Tan et al., 2021). In addition, interferon-gamma (INF-γ) released from activated CD8^+^ T cells could promote lipid peroxidation, resulting in the ferroptosis of tumor cells (Wang et al., 2019). Besides, ferroptotic tumor cells further inhibit the tumor-suppressing function of cytotoxic T cells and NK cells through releasing prostaglandin E2 (PGE2) (Yang et al., 2014; Johnson et al., 2020; Xu et al., 2021). Moreover, n-3 PUFA docosahexaenoic acid (DHA), which was reported to cause ferroptosis of tumor cells, could also induce the pyroptosis in tumor cells, suggesting that lipid metabolism might well be the junction point between ferroptosis and pyroptosis (Ou et al., 2017; Pizato et al., 2018; Du et al., 2019). Considering that the process of impaired plasma membrane occurred in both cell death types, underlying connections between ferroptosis and pyroptosis remain intriguing for investigation. Though recently published study has explored the role of ferroptosis-related genes in prognosis and immune activity of PAAD, the lack of analyzing pyroptosis-related gene is insufficient to reflect the crosstalk between cell death subtypes and tumor biology (Tang et al., 2020a). Currently, the extent to which pyroptosis and ferroptosis influences the tumor biology remains ambiguous, especially in PAAD that characterized with “cold” immunity. Developing translational strategies against PAAD depends on better understanding about the complicated roles and related signaling pathways of pyroptosis and ferroptosis.

In this study, four robust cell death subtypes of PAAD were identified based on consensus clustering of pyroptosis- and ferroptosis-related gene expression profiles, which were associated with distinct survival, mutational, and immune signatures. Furthermore, the pyroptosis-ferroptosis (P-F) score was developed, with superior capacity in predicting the outcomes and responses of patients to chemotherapeutic agents and immune checkpoint blockades (ICBs) (Supplementary Figure S1).

## Materials and Methods

### Data Extraction and Data Processing

The RNA-sequence (RNA-Seq) data with matched clinical information of all available PAAD patients were extracted from The Cancer Genome Atlas (TCGA) (https://www.cancer.gov/tcga) (TCGA-PAAD, n = 176) and the International Cancer Genome Consortium (ICGC) (https://daco.icgc.org/) (ICGC-PACA-CA, n = 165) databases. For subsequent analyses, fragments per kilobase million (FPKM) values were converted to transcripts per kilobase millions (TPMs)-normalized. Additionally, the E-MTAB-6134 dataset with complete clinical information of 288 PAAD patients and datasets without detailed clinical information including GSE57495 based on platform GPL15048, GSE21501 based on platform GPL4133, and GSE85916 based on platform GPL13667 were all extracted from the Array Express database (https://www.ebi.ac.uk/arrayexpress). The raw data of the gene expression in E-MTAB-6134, GSE57495, GSE21501, and GSE85916 datasets were normalized by using the “limma” R package. The TCGA-PAAD dataset was utilized as training cohort, and the other datasets were set as validation cohorts. Based on the Creative Commons 3.0 license that was downloaded from http://research-pub.gene.com/IMvigor210CoreBiologie, the IMvigor210 dataset was extracted from a freely available data package. The IMvigor210 dataset containing 298 patients of urothelial cancer who had received immunotherapy was performed to validate the prediction value of the P-F score. The corresponding information of somatic mutations in TCGA-PAAD patients was extracted from UCSC Xena (https://xena.ucsc.edu/). Somatic mutations were analyzed and visualized through the “maftool” R package (Mayakonda et al., 2018). Furthermore, the copy number variations (CNVs) of TCGA-PAAD patients were visualized and presented by using the “RCircos” package.

### Cell Death Subgrouping

Through referring to previously published reviews and relevant bioinformatic study, we extracted 33 pyroptosis-related genes (Man and Kanneganti, 2015; Wang and Yin, 2017; Kambara et al., 2018; Kang et al., 2018; Karki and Kanneganti, 2019; Xia et al., 2019; Li et al., 2021; Wang et al., 2021). Considering that some bioinformatic studies and basic studies have identified extra pyroptosis-related genes, we verified the function of these genes in the HUMAN GENE database (https://www.genecards.org/) and further extracted another 6 pyroptosis-related genes (Zhu et al., 2017; Dong et al., 2021; Ju et al., 2021; Zhang et al., 2021). By taking the union, a total of 39 pyroptosis-related genes were extracted and listed in Supplementary Table S1. Moreover, 113 ferroptosis-related genes were also identified mainly based on previously published reviews and relevant bioinformatic studies (Supplementary Table S2) (Stockwell et al., 2017; Bersuker et al., 2019; Doll et al., 2019; Hassannia et al., 2019; Liang et al., 2020; Liu et al., 2020; Zhuo et al., 2020). Then, consensus clustering was carried out based on pyroptosis- and ferroptosis-related genes through the “ConsensusClusterPlus” R package, with the repeats of 1,000, pItem of 0.8, and pFeature of 1 to guarantee the stability of classification. With max k = 5, the Ward. D2 and Pearson correlations were separately served as the clustering algorithm and distance metric. Next, each sample was assigned into the quiescent (pyroptosis ≤0, ferroptosis ≤0), pyroptosis (pyroptosis >0, ferroptosis ≤0), ferroptosis (pyroptosis ≤0, ferroptosis >0), and mixed (pyroptosis >0, ferroptosis >0) subtypes according to the median expression levels of co-expressed pyroptosis- and ferroptosis-related genes.

### Collection of Immune-Related Data

Based on evaluating the LM22 signature, the immune cell infiltration in each sample of the TCGA-PAAD cohort was analyzed using the “CIBERSORT” R package (Newman et al., 2015). Besides, the ESTIMATE algorithm was applied to calculate the ESTMATE, immune, and stromal scores in each PAAD sample (Yoshihara et al., 2013).

### Dimension Reduction and Construction of the P-F Score

According to the expression levels of identified genes associated with cell death patterns, the PAAD patients were assigned into corresponding subtypes. Then, the differentially expressed genes (DEGs) across these subtypes were screened by using the “limma” R package, setting the cutoff values as |log_2_ fold change (FC)| > 1 and *p* < 0.05 (adjusted). The R package of “clusterProfiler” was performed for Gene Ontology (GO) enrichment analysis. Next, unsupervised clustering analysis was performed to stratify the patients of the TCGA-PAAD cohort into distinct gene clusters according to their DEG values. The DEGs that positively correlated to the cluster signature were referred to as P-F gene signature A, while the residual DEGs were referred to as P-F gene signature B. To get rid of the noise or redundant genes, the dimension reduction of the P-F gene signatures A and B was further conducted by using the Boruta algorithm (Kursa and Rudnicki, 2010). Moreover, the principal component analysis (PCA) algorithm was used for extracting principal component 1 as the signature score. Finally, referring to the gene expression grade index, the P-F score of each sample was calculated according to the following equation: P-F score = ∑PC1_A_ - ∑PC1_B_ PC1_A_ stands for the first component of signature A, and PC1_B_ stands for the first component of signature B.

### Prediction of Therapeutic Benefits in Patients With Distinct P-F Scores

To better predict the response to ICBs in cancer patients, the Tumor Immune Dysfunction and Exclusion (TIDE) algorithm was developed as a computational method to model the primary mechanisms of tumor immune escape (Jiang et al., 2018). Therefore, the TIDE web application (http://tide.dfci.harvard.edu) was utilized to evaluate the utility of the P-F score in predicting the therapeutic response to ICBs for PAAD patients. Subsequently, the subclass mapping algorithm (https://cloud.genepattern.org/gp/) was implemented to visualize the therapeutic responses to anti-PD-1 and anti-CTLA4 therapeutics between distinct subgroups based on previously reported 47 melanoma patients with detailed immunotherapy records (Roh et al., 2017). Furthermore, sensitivity to chemotherapeutic agents including gemcitabine, cisplatin, paclitaxel, and 5-fluorouracil was estimated by using the R package of “pRRophetic,” which was based on the Genomics of Drug Sensitivity in Cancer (GDSC). To compare the drug sensitivity between the high and low P-F score groups, the estimated half-maximal inhibitory concentration (IC50) of each sample was computed with the ridge regression, and tenfold cross-validation was employed to evaluate the accuracy of this prediction (Geeleher et al., 2014).

### Connectivity MAP Analysis

As a public online tool, the Connectivity MAP database (CMap, https://portals.broadinstitute.org/cmap/) enables the user to predict small molecules that could target cancer-related genes based on gene expression profiles (Lamb et al., 2006). To predict potential small molecular drugs for PAAD, the DEGs between the high and low P-F score groups were identified and input into the CMap database. Besides, the CMap mode-of-action (MoA) analysis was performed in order to reveal the underlying mechanism of drug actions (Subramanian et al., 2017).

### Statistical Analysis

All statistical analyses in this study were conducted using the R software (version 4.0.4). Comparisons between two groups or more than two groups were conducted through the Wilcoxon test or Kruskal–Wallis test, respectively. Chi-square test was used for analyzing the correlations between categorical variables. The correlation coefficient was calculated through Spearman analysis. Survival analysis for each dataset was conducted using the Kaplan–Meier plotter, where the statistical difference was evaluated through the log rank test. To estimate the predictive efficacy of the variate, time-dependent analysis of the receiver operating characteristic (ROC) curve was performed to calculate the area under the curves (AUCs) through the R package “survivalROC.” The R package “survival” was used for univariate and multivariate Cox regression analyses. Unless stated otherwise above, the statistical significance was considered with a two-tailed *p* < 0.05.

## Results

### Stratify the Cell Death Subtypes of PAAD Based on Dual Analysis of Pyroptosis- and Ferroptosis-Related Genes

**FIGURE 1:**
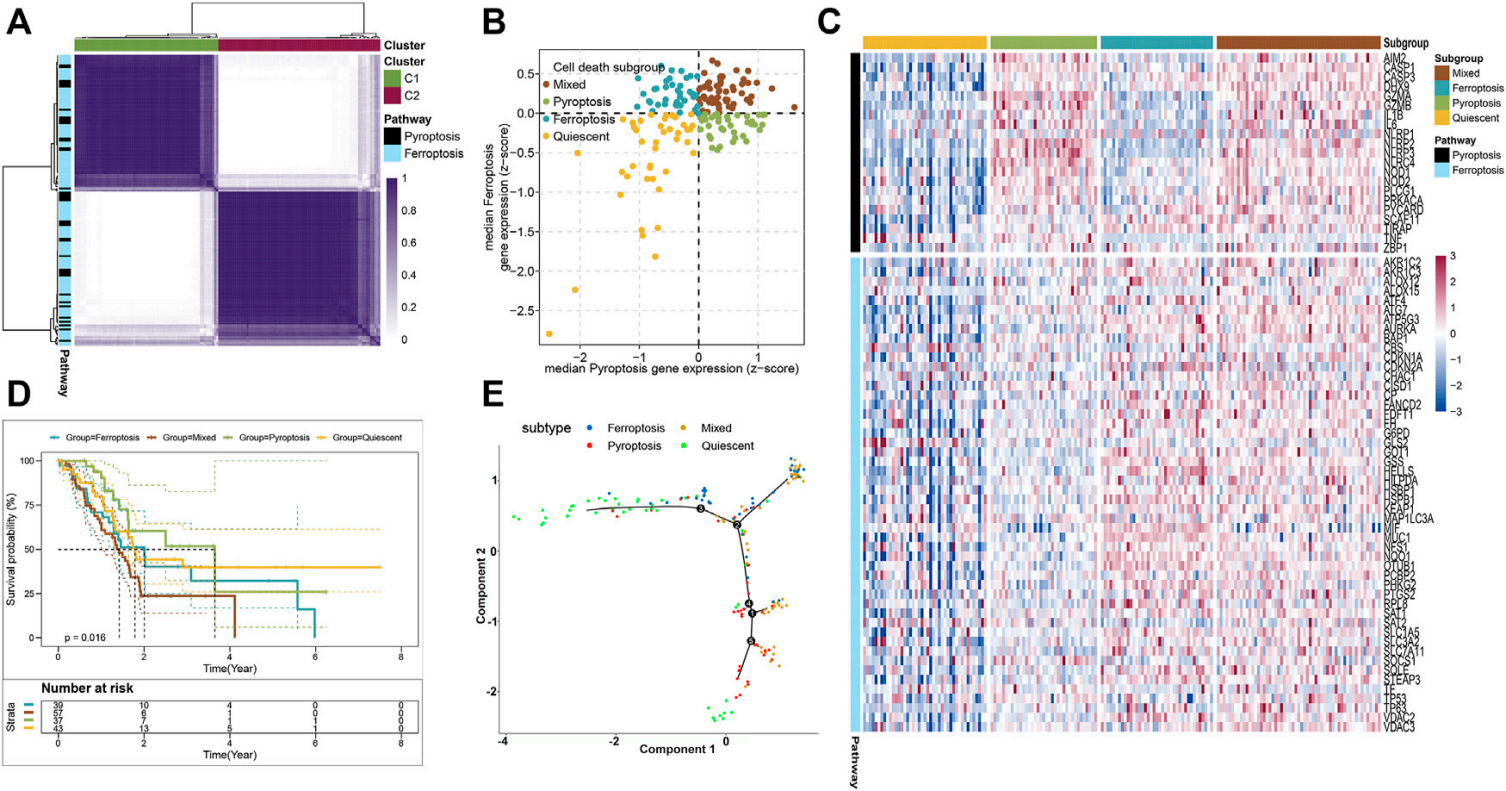
Stratifying the cell death subgroups of PAAD according to the expression profile of pyroptosis- and ferroptosis-related genes. **(A)** Clustering heatmap of the pyroptosis- and ferroptosis-related genes in the TCGA-PAAD cohort (n = 176). **(B)** As shown in the scatter plot, the PAAD patients were stratified into 4 cell death subtypes based on the median expression levels of coexpressed pyroptosis- (*x*-axis) and ferroptosis-related genes (*y*-axis). **(C)** Heatmap demonstrated the expression status of the pyroptosis- and ferroptosis-related genes among each subtype. **(D)** Survival analysis of the PAAD patients with different cell death subtypes (log-rank *p* = 0.016). **(E)** The distributions of the four subtypes using dimensionality reduction analysis. Each point represented a patient, with the color corresponding to the previously defined cell death subtypes.

To stratify the cell death subtypes of PAAD, the RNA-sequence data from the TCGA-PAAD cohort were analyzed with their expression levels of pyroptosis and ferroptosis pathway genes. As shown in Figure 1A, the consensus cluster plus was applied to identify the groups that mainly coexpressed pyroptosis and ferroptosis pathway genes for cell death subgrouping. The median expression levels of coexpressed pyroptosis- and ferroptosis-related genes in each PAAD sample were computed. The PAAD samples were then assigned into the cell death subtypes particularly corresponding to these two pathways: quiescent, pyroptosis, ferroptosis, and mixed (Figure 1B). The heatmap displayed the expression profiles of the pyroptosis- and ferroptosis-related genes across these four subtypes (Figure 1C and Supplementary Figure S2). The patients in the mixed subtype were significantly relevant to the worst survival, while a relatively better survival was found in patients of the pyroptosis subtype (Figure 1D). To better visualize the distribution of individual patients, dimensionality reduction analysis was utilized through analyzing pyroptosis- and ferroptosis-related gene expression profiles. The locations of individual patients were assigned into the tree structure, which indicated the differences among the four subtypes (Figure 1E). Collectively, these findings demonstrated four distinct cell death subtypes associated with the pyroptosis–ferroptosis pathway in PAAD, in which the tumors that coexpressed with higher levels of pyroptosis- and ferroptosis-related genes were associated with worse prognosis. We further investigated the correlations between the cell death subtypes and TIME. The pyroptosis subtype exhibited significantly higher infiltrations of activated CD4^+^ memory T cells and CD8^+^ T cells, while the mixed subtype showed the lowest infiltration of activated NK cells (Supplementary Figure S3A). Besides, the expression levels of immune checkpoints (ICPs)-related genes and immunogenic cell death (ICD)-related genes also showed distinct differences across the four subtypes (Supplementary Figures S3B, C). Given that the “limma” R package could only compare two groups at a time, the DEGs across the four subtypes were the summation of DEGs identified between two subtypes by using the “limma” R package, setting the cutoff values as |log_2_ FC| > 1 and *p* < 0.05 (adjusted). A total of 6,164 DEGs across the four subtypes were then identified (Supplementary Table S3), and the GO functional enrichment of these DEGs was mainly enriched in the biological process (BP) involved in the T-cell activation, positive regulation of cell adhesion, and leukocyte cell–cell adhesion (Supplementary Figure S3D and Supplementary Table S4).

**FIGURE 2:**
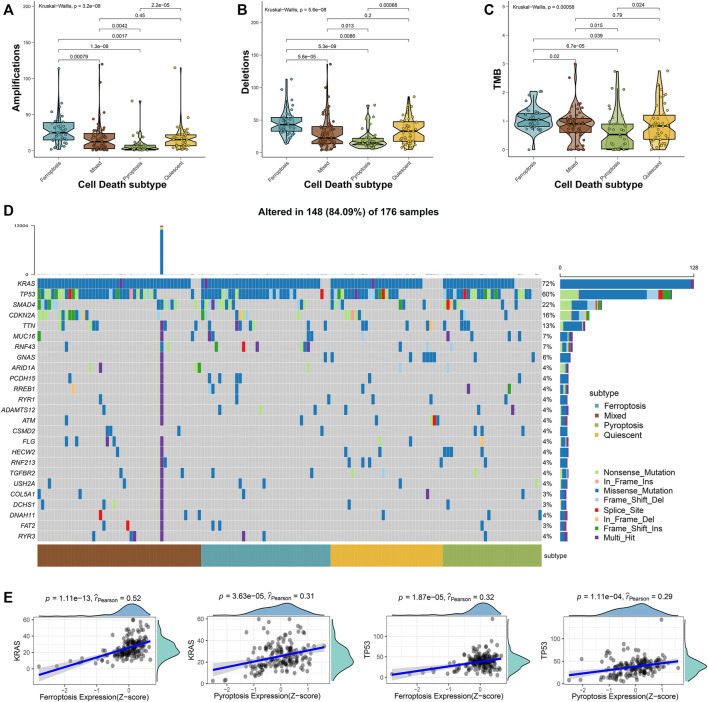
Mutational landscape across the cell death subtypes in PAAD samples. The amplications **(A)**, deletions **(B)**, and TMB **(C)** were evaluated across the 4 cell death subtypes. **(D)** The oncoplot depicted the top 25 most frequently mutated genes across the subgroups of PAAD samples. **(E)** The correlations between the expression of ferroptosis- or pyroptosis-related genes and *KRAS* and *TP53* expressions.

Approximately 95% of the PAAD patients have gene alterations, including mutations, amplifications, deletions, or inversions (Escobar-Hoyos et al., 2020). Therefore, the mutational landscape was investigated among the 4 cell death subtypes. The ferroptosis subtype exhibited significantly highest gene alterations involved in amplifications, deletions, and tumor mutation burden (TMB) (Figures 2A–C). Besides, the mutational landscape of the top 25 most frequently mutated genes was evaluated in PAAD, which demonstrated obviously maximum mutations in the mixed subtype (Figure 2D). We further observed a strong correlation between the expressions of ferroptosis-related genes with *KRAS* (Spearman coefficient R = 0.52, *p* = 1.11e-13) and a moderate correlation with *TP53* (Spearman coefficient R = 0.32, *p* = 1.87e-5), while the expression of pyroptosis-related genes showed a moderate correlation with the *KRAS* expression (Spearman coefficient R = 0.31, *p* = 3.63e-5) and a weak correlation with the *TP53* expression (Spearman coefficient R = 0.29, *p* = 1.11e-4) (Figure 2E). The relatively worse survival of patients in ferroptosis and mixed subtypes was compatible with the notion that the higher mutation accumulation in cancer has negative correlation with the overall survival (OS) of patients (Pleasance et al., 2020). These results implied that ferroptosis and pyroptosis might cooperate to promote the progression of PAAD, even when accounting the better survival in patients of the pyroptosis subtype. Thus, a comprehensive classification about distinct cell death profiles was needful for better stratifying the PAAD patients.

### Identification of the P-F Score in TCGA-PAAD Training Dataset

**FIGURE 3:**
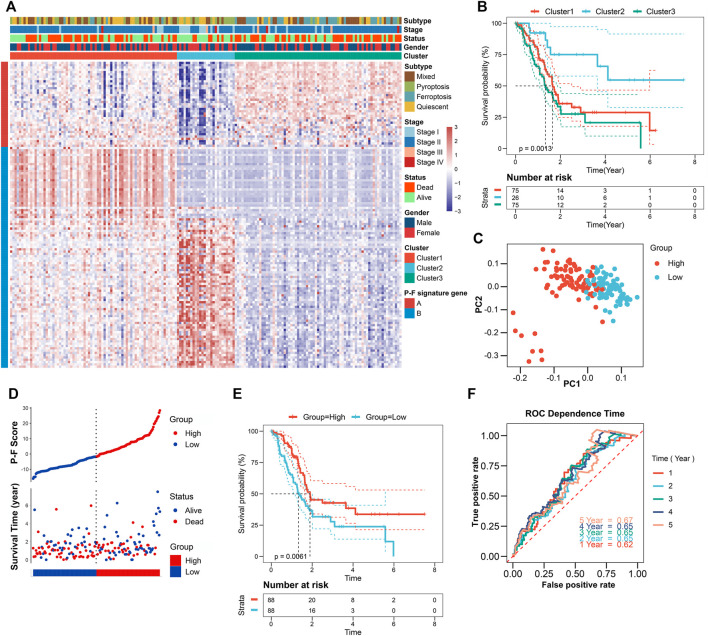
Identification of the P-F score in the TCGA-PAAD training dataset. **(A)** Unsupervised clustering of the DEGs among the 4 cell death subtypes to further stratify PAAD patients into three gene clusters. **(B)** Survival analysis of the PAAD patients in three gene clusters (log-rank *p* = 0.0013). **(C)** Principal components analysis (PCA) of the PAAD patients with high or low P-F scores. **(D)** Distribution of the P-F score and survival status of PAAD patients. **(E)** Survival analysis of the PAAD patients in high and low P-F score groups (log-rank *p* = 0.0061). **(F)** ROC curves for the 1-, 2-, 3-, 4-, and 5-year survival times based on the P-F score.

To reveal the underlying biological behaviors of these cell death subtypes, differential expression analysis was performed. Based on the 6,164 DEGs identified from previous differential analysis, the unsupervised clustering stratified the TCGA-PAAD cohort into three genomic clusters, named clusters 1–3 (Supplementary Figure S4). The 2,602 DEGs that have positive correlations with the gene cluster were referred to as P-F gene signature A, and the residual DEGs were referred to as P-F gene signature B (Supplementary Table S5). To get rid of the noise or redundant genes, the dimension reduction of the P-F gene signatures A and B was further conducted through the Boruta algorithm (Kursa and Rudnicki, 2010). The transcriptomic profile of the 133 most abundant DEGs identified among the genomic clusters was displayed by the heatmap (Figure 3A and Supplementary Table S6). When compared with other genomic clusters, the patients in cluster 2 exhibited the significantly longest OS, while the patients in clusters 1 and 3 had relatively poorer prognosis (Figure 3B). Concurrently, for the purpose of achieving a quantitative indicator of the P-F landscape in PAAD patients, PCA was used to calculate the integrated scores, including PC1_A_ from P-F gene signature A (n = 37) and PC1_B_ from P-F gene signature B (n = 96). The individual score of each patient was calculated from the integration of corresponding PC1_A_ and PC1_B_. Finally, the obtained signature score was defined as the P-F score. Thereafter, the patients in the TCGA-PAAD cohort were divided into high and low P-F score groups based on the corresponding median value of P-F scores. Furthermore, PCA of individual patients has shown a fine distinction between the high and low P-F score groups, which further confirmed that our P-F scoring system could distinguish the PAAD patients well (Figure 3C). The prognostic value of the P-F score was evaluated. The survival status of individual patients was depicted by the scatter plot (Figure 3D). The patients in the high score group showed a significantly longer OS (log-rank *p* = 0.0061; Figure 3E). The AUCs were 0.62, 0.65, 0.65, 0.65, and 0.67 for 1-, 2-, 3-, 4-, and 5-year survival times, respectively, indicating the reliability of the P-F score for predicting the outcomes of PAAD patients (Figure 3F). Moreover, the prognostic efficiency of the P-F score was also validated in the ICGC-PACA-CA cohort (Supplementary Figure S5), the E-MTAB-6134 cohort (Supplementary Figure S6), GSE57495, GSE21501, and GSE85916 (Supplementary Figure S7), which have shown similar results.

### Prognostic Value of the P-F Score for PAAD Patients

**FIGURE 4:**
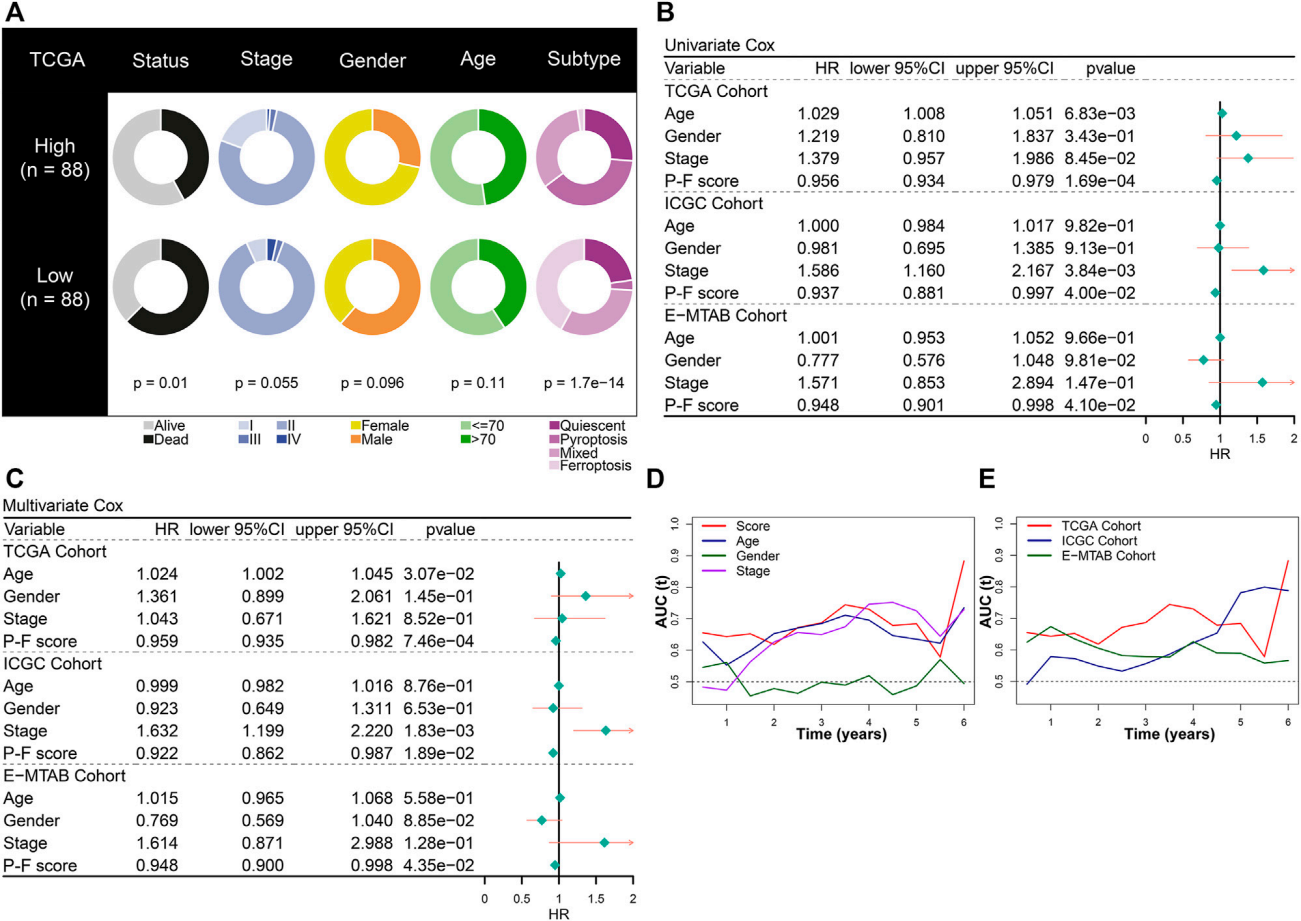
The prognostic value of the P-F score in PAAD. **(A)** Constitution of the clinical features and cell death subtypes in the high and low P-F score groups. Both univariate **(B)** and multivariate **(C)** Cox regression analyses suggested that the P-F score was a significant prognostic factor for OS in the training (TCGA-PAAD) and validation (ICGC-PACA-CA and E-MTAB-6134) cohorts. **(D)** The time-dependent AUC values of P-F score, age, gender, and stage for the OS prediction in the training cohort (TCGA-PAAD). **(E)** The time-dependent AUC values of the P-F score for the OS prediction in the training and validation cohorts.

The prognostic value of the P-F score was further explored in depth. The constitution of clinical features and cell death subtypes in high and low P-F score groups from the TCGA-PAAD cohort is depicted in Figure 4A. No significant difference in stage, gender, and age was found between the high and low score groups, while the low score group showed a significantly worse survival status (*p* = 0.01). Besides, the distribution of cell death subtypes showed a significant difference, with the pyroptosis subtype mainly distributed in the high score group and the ferroptosis subtype mainly in the low score groups (*p* = 1.7e-14). Both in the training cohort (TCGA-PAAD) and the validation cohort (ICGC-PACA-CA and E-MTAB-6134) the P-F score was identified as an independent protective factor for PAAD, based on the univariate and multivariate Cox regression analyses (Figures 4B,C). According to time-dependent AUC values, the P-F score displayed a well predictive power for OS in comparison with that of age, gender, or stage in the TCGA-PAAD cohort (Figure 4D). The excellent predictive ability of the P-F score for OS was also explored in ICGC-PACA-CA and E-MTAB-6134 cohorts (Figure 4E). Furthermore, stratified analysis has also shown a significant difference in the OS between the high and low score groups with distinct stage, gender, and age (Supplementary Figure S8). Taken together, these results suggested that the P-F score could serve as an independent protective factor for PAAD patients.

### The Correlation Between the P-F Score and TIME

**FIGURE 5:**
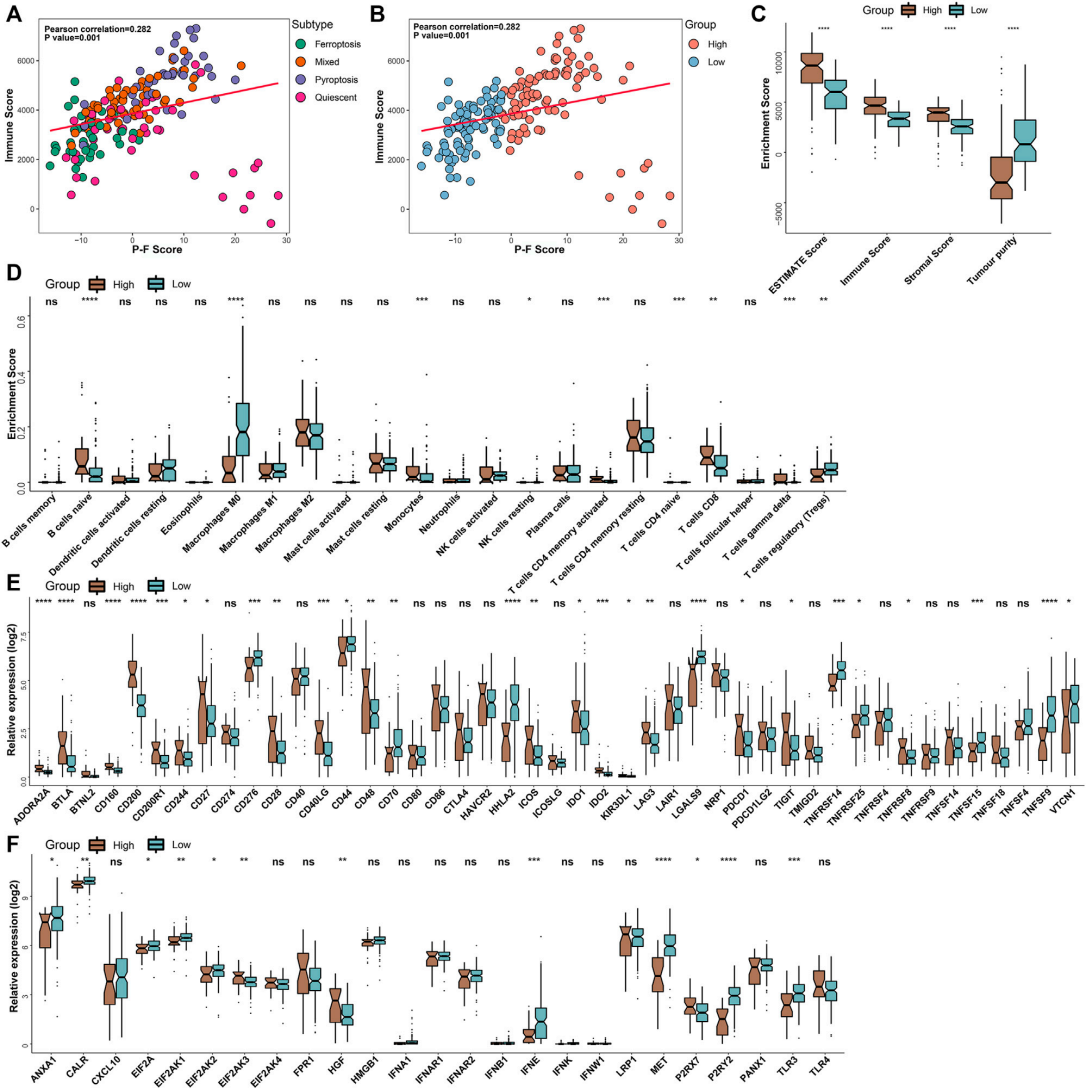
Tumor immunological analyses in the P-F score subgroups. The scatter plots depicted the correlation between the immune score and the P-F score of tumor samples corresponding to 4 cell death subtypes **(A)** and P-F score groups **(B)** in the TCGA-PAAD cohort. **(C)** Enrichment scores of the ESTIMATE score, immune score, stromal score, and tumor purity in the high and low P-F score groups. **(D)** Box plots demonstrated the infiltration levels of multiple immune cells between the distinct P-F score groups. Evaluating the expression levels of the ICPs-relevant genes **(E)** and ICDs-relevant genes **(F)** between the distinct P-F score groups (ns, not significant; *p < 0.05; **p < 0.01; ***p < 0.001; and ****p < 0.0001).

To further illuminate intrinsic biological diversities that contributed to the distinct survival status, the correlation between the P-F score and the infiltration of immune components was investigated. The correlation analyses indicated that the P-F score was significantly and positively related to the immune score (Spearman coefficient R = 0.282, *p* = 0.001) (Figures 5A,B). Meanwhile, the high P-F score group demonstrated significantly higher levels of ESTIMATE score, immune score, and stromal score, while the higher tumor purity was found in the low P-F score group (Figure 5C). Furthermore, the relation between tumor immunological status and the P-F score was evaluated. The high P-F score group exhibited significantly escalated infiltration of activated CD4^+^ memory T cells, CD8^+^ T cells, and gamma-delta (γδ) T cells, which was characterized with the active immune phenotype. The low P-F score group was associated with significantly higher infiltration of immunosuppressive regulatory T cells (Tregs) (Figure 5D). The active immune phenotype characterized with abundant T-cell infiltration and enhanced cytolytic activity was correlated with better outcomes, just as we have observed in the high P-F score group (Chen et al., 2019). Moreover, the expression levels of the ICPs- and ICDs-related genes also showed significant differences between the two groups. Among the 28 differentially expressed ICPs-related genes, a total of 18 (18/28, 64.2%) genes were significantly upregulated in the high P-F score group than the low P-F score group (Figure 5E). The low P-F score group exhibited significantly higher expression levels of the ICDs-related genes (9/12, 75%) (Figure 5F). Therefore, we speculated from these results that the high P-F score group with higher expressions of ICPs-related genes might benefit more from the ICBs therapy, while the low P-F score group with higher expressions of ICDs-related genes might benefit more from the ICDs-based cancer vaccines (Jin and Wang, 2021). The consistency between the immune phenotype and prognosis status in these two P-F score subgroups further demonstrated the scientificity and reliability of our classification strategy.

### Functional Enrichment Analysis of the DEGs in the P-F Score Groups

**FIGURE 6:**
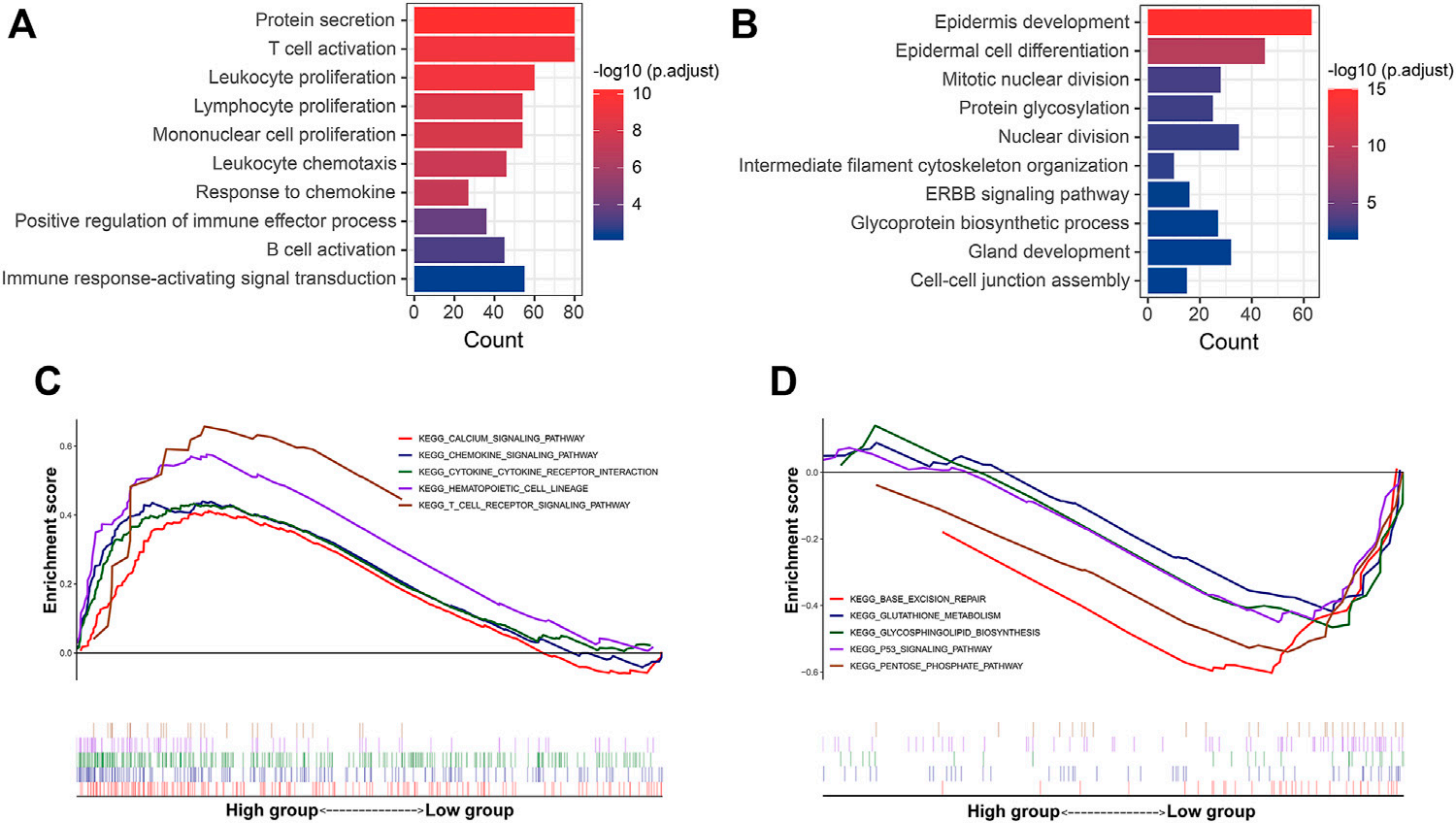
Functional enrichment analyses of the DEGs between the high and low P-F score groups. The GO enrichment analysis of the upregulated **(A)** and downregulated **(B)** DEGs in the high P-F score group when compared with the low group. GSEA showing the pathways enriched in the high **(C)** and **(D)** low P-F score groups.

Additionally, functional enrichment analysis was performed to elucidate the bioinformatic functions of the DEGs between the P-F score groups. Compared with the low P-F score group, the upregulated DEGs in the high P-F score group were mainly enriched in the activation and proliferation of immune cells, inflammatory response, chemokine, and positive regulation of immune effector process, while the downregulated DEGs were mainly enriched in the mitotic nuclear division, nuclear division, and ERBB signaling pathway (Figures 6A,B and Supplementary Tables S7, S8). Moreover, Gene Set Enrichment Analysis (GSEA) revealed that the chemokine signaling pathway, cytokine–cytokine receptor interaction, and T-cell receptor signaling pathway were significantly enriched in the high P-F score group, whereas the p53 signaling pathway was enriched in the low P-F score group (Figures 6C,D and Supplementary Tables S9, S10). Consistently with the immune profiles, the high P-F score was mainly relevant to the active immune phenotype and better outcome, while the low P-F score group was relevant to the phenotype of more active cell proliferation and worse outcome.

### Correlations Between the P-F Score and Somatic Mutations

**FIGURE 7:**
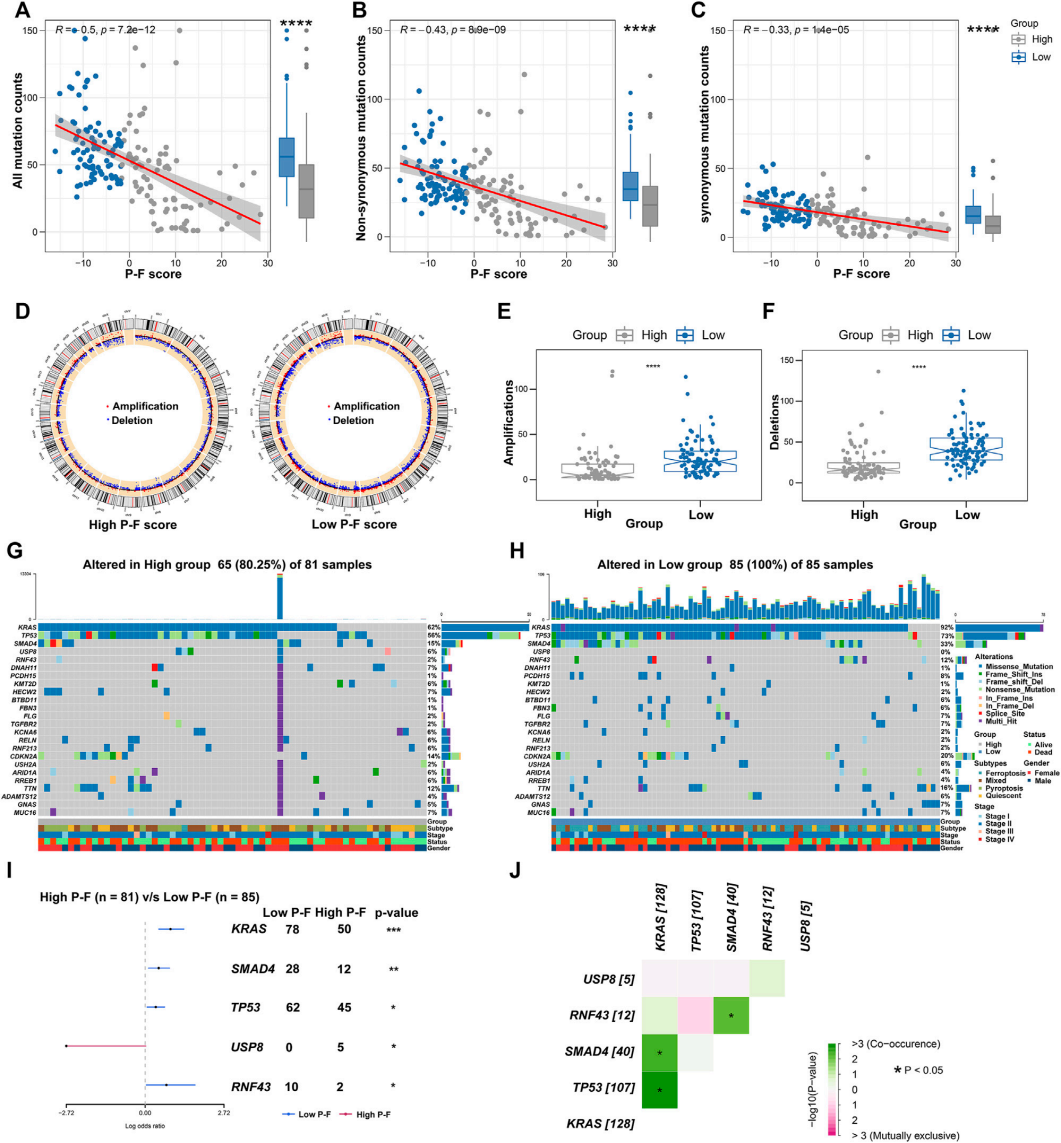
Correlations between the P-F score and tumor mutation status. Correlations between all mutation counts **(A)**, non-synonymous mutation counts **(B)**, synonymous mutation counts **(C)**, and P-F score, and their distributions in the distinct score groups. **(D)** Circos plots depicted the locations of CNV alteration in the high and low P-F score groups. The red dots indicated the amplifications, and the blue dots indicated the deletions. The amplifications **(E)** and deletions **(F)** of chromosome were evaluated in distinct score groups. The oncoplot depicted the landscape of 24 genes with most frequently genomic alteration in the high **(G)** and low **(H)** score groups. **(I)** Forest plot showed the mutated genes that were significantly different between the two groups. **(J)** Interactions among the mutated genes that were significantly different between the high and low P-F score groups (*p < 0.05; **p < 0.01; ***p < 0.001; and ****p < 0.0001).

Given the significant value of genomic alterations in regulating tumor immunity, analyses about the somatic mutation and CNVs were performed to investigate the genomic alterations between the P-F score subgroups (Rooney et al., 2015). As demonstrated in Figure 7A, the P-F score was significantly and negatively associated with all mutation counts (Spearman coefficient R = 0.5, *p* = 7.2e-12). Compared with the patients in the high P-F score group, the patients in the low P-F score group have shown significantly elevated somatic mutations, including the non-synonymous and synonymous mutations (Figures 7B,C). Besides, CNV analysis has exhibited significantly improved copy number amplifications and deletions in the low P-F score group than those in the high P-F score group (Figures 7D–F). Additionally, the top 24 genes with the most frequently genomic alteration in PAAD were analyzed between the high and low P-F score groups. The patients in the low P-F score group have showed more frequently genomic alterations than those in the high P-F score group (Figures 7G,H). As the most frequently oncogenic mutation in PAAD, the proportion *KRAS* mutations in the low P-F score group (92%) was remarkably higher than that in the high P-F score group (62%). Meanwhile, the alterations of *TP53*, *SMAD4*, *USP8*, and *RNF43* also were significantly distinct between the high and low P-F score groups (Figure 7I). Among these mutated genes, the *KRAS*, *TP53*, *SMAD4*, and *RNF43* exhibited significant co-occurrence (Figure 7J). Taken together, these results revealed the relationships of the P-F score and genomic alteration in PAAD, in which the patients in the low P-F score group manifested with remarkably more alterations.

### Role of the P-F Scores in Predicting Therapeutic Benefits

**FIGURE 8:**
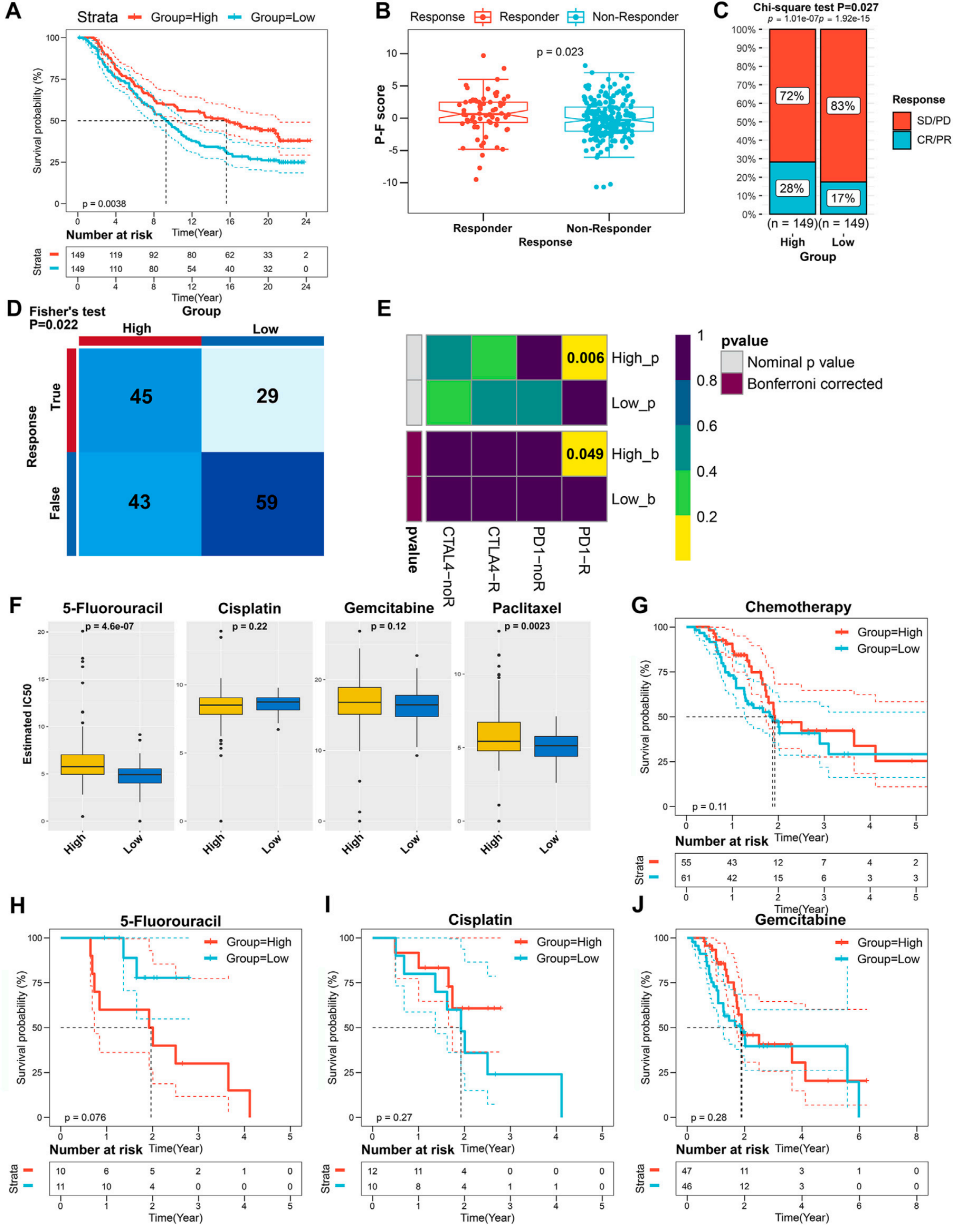
The role of the P-F score in predicting the benefits from immunotherapy and chemotherapy. **(A)** Kaplan–Meier curve for patients with distinct P-F scores from the IMvigor210 cohort (log-rank *p* = 0.0038). **(B)** Evaluation of the P-F scores among patients with distinct anti-PD-L1 treatment responses (Wilcoxon test *p* = 0.023). **(C)** Clinical responses to the anti-PD-L1 immunotherapy of patients in high or low P-F score groups from the IMvigor210 cohort (SD, stable disease; PD, progressive disease; CR, complete response; PR, partial response). **(D)** The responses to anti-PD-1 and anti-CTLA4 immunotherapies of PAAD patients with high or low P-F scores in the TCGA-PAAD cohort were predicted by using the TIDE algorithm (Fisher’s test *p* = 0.022). **(E)** Heatmap visualized the responses to anti-PD-1 and anti-CTLA4 immunotherapies between the distinct P-F score groups. **(F)** The estimated IC50 levels of 5-fluorouracil, cisplatin, gemcitabine, and paclitaxel between the two groups. **(G)** Kaplan–Meier curve for patients who have received chemotherapy in the TCGA-PAAD cohort (log-rank *p* = 0.11). Kaplan–Meier curves for patients in the TCGA-PAAD cohort who have been treated with 5-fluorouracil **(H)** (log-rank *p* = 0.076), cisplatin **(I)** (log-rank *p* = 0.27), or gemcitabine **(J)** (log-rank *p* = 0.28).

A myriad of studies has validated the great potential of ICB therapy in various cancer types (Sharma and Allison, 2015). However, given the complexity of the TIME, not all cancer patients could respond to ICB therapy (Brahmer et al., 2012; Powles et al., 2014). Therefore, we next explored the availability of the P-F score in predicting the benefits from immunotherapy. First, cancer patients from the IMvigor210 cohort who have received anti-PD-L1 immunotherapy were allocated into the high or low P-F score group based on P-F scoring. As shown in the Kaplan–Meier curve, the patients in the high P-F score group exhibited a significantly better survival when compared with the patients in the low P-F score group (Figure 8A). Notably, it was a phenomenon in which significantly more responders after anti-PD-L1 treatment were observed in the high P-F score group (Figure 8B). Additionally, the high P-F score group has shown a complete response/partial response (CR/PR) rate of 28% to anti-PD-L1 immunotherapy in the IMvigor210 cohort, which was remarkably higher than that in the low P-F score group (17%) (Figure 8C). Encouraged by the abundant T lymphocytes infiltration in the high P-F score group, we next speculated the response to ICBs through the TIDE algorithm. As expected, the patients in the high P-F score group had a significantly higher response rate to the ICBs than the low P-F score group in the TCGA-PAAD cohort (Fisher’s test *p* = 0.022) (Figure 8D). Furthermore, a subclass mapping algorithm was performed to visualize the therapeutic responses based on the previously reported 47 melanoma patients with detailed immunotherapy records. The heatmap depicted that patients in the high P-F score group were more responsive to the anti-PD-1 immunotherapy in the TCGA-PAAD cohort (Bonferroni corrected *p* = 0.049) (Figure 8E). The consistent phenomenon was also validated in the ICGC-PACA-CA and E-MTAB-6134 cohorts (Supplementary Figures S9, S10).

Currently, the systemic chemotherapy regimens including FOLFIRINOX or gemcitabine plus paclitaxel are still the mainstay of therapy for PAAD patients (Mizrahi et al., 2020). Therefore, we subsequently investigated the utility of the P-F score in speculating the response to the commonly used chemotherapeutic agents. As shown in Figure 8F, the estimated IC50 levels of paclitaxel and 5-fluorouracil were significantly lower in the low P-F score group than those in the high P-F score group, which indicated that the patients in the low P-F score group might be more sensitive to these two drugs. Besides, significantly decreased estimated IC50 levels of paclitaxel, 5-fluorouracil, and gemcitabine were noticed in the low P-F score group in the ICGC-PACA-CA and E-MTAB-6134 cohorts, respectively (Supplementary Figure S9C and Supplementary Figure S10C). Given the lack of therapeutic data in the ICGC-PACA-CA, E-MTAB-6134, GSE57495, GSE21501, and GSE85916 datasets, we only stratified the patients from the TCGA-PAAD cohort based on distinct chemotherapeutic regimens to evaluate whether the P-F score determined differently in survival. Subsequently, the patients who have received chemotherapy in the TCGA-PAAD cohort were assigned based on the high and low P-F scores. There was no significant difference observed in survival between the high and low P-F score groups (Figure 8G). In the subgroup of patients who had been treated with 5-fluorouracil, cisplatin, or gemcitabine, the Kaplan–Meier curves also showed no statistically significant difference in survival between the high and low P-F score groups (Figures 8H–J). When treated with 5-fluorouracil, the patients in the low P-F score group tend to have relatively better survival than those in the high P-F score group, though no statistically significant difference was observed (log-rank *p* = 0.076). These results together suggested that the patients with high P-F scores might be more responsive to immunotherapy, while the patients with low P-F scores might benefit more from chemotherapeutic agents such as paclitaxel or 5-fluorouracil. Because of the limited number of patients who have been treated with 5-fluorouracil and paclitaxel in the TCGA-PAAD cohort, further large-scale clinical trials are urgently required to investigate the prognostic value of the P-F score in the chemotherapy for PAAD. Based on the value of |log_2_ FC|, the top 150 upregulated DEGs and 150 downregulated DEGs between the high and low P-F score groups were uploaded to the CMap small molecular drug database to search the underlying drugs. As shown in Supplementary Figure S11, a total of 55 potential small molecular drugs and 30 drug mechanisms were identified, in which the HDAC inhibitors accounted for the highest proportion among the inhibitors (Supplementary Table S11). Taken together, these findings have highlighted the potential value of the P-F score in selecting more suitable therapeutic strategy for PAAD patients.

## Discussion

To facilitate the development of individualized therapy for PAAD patients, persistent progress in classifying clinically relevant subtypes of PAAD is urgently needed. In this study, we have established a scoring system (P-F score) based on the consensus clustering of pyroptosis- and ferroptosis-related gene expression profiles in PAAD patients. The results of this study indicated that the P-F score could work as a reliably independent prognostic factor and predictive indicator to estimate the therapeutic responses to immunotherapy and chemotherapy. The PAAD patients with high P-F scores were characterized with escalated immune and stromal score, active immune phenotype, lower tumor purity, and genomic alterations when compared with those in patients with low P-F scores. Furthermore, the patients in the high P-F score group were more responsive to the anti-PD-L1 representative immunotherapy, while the patients in the low P-F score group might be more sensitive to the chemotherapeutic agents like paclitaxel or 5-fluorouracil.

Currently, the exploration of non-apoptotic cell death processes has accelerated the advances in treating malignancies (Chen et al., 2021a). Pyroptosis and ferroptosis have shown complex effects in the biology and therapy of cancer that vary in genetic backgrounds (Friedmann Angeli et al., 2019; Chen et al., 2021b; Wu et al., 2021). Although the roles of pyroptosis and ferroptosis in tumor biology and antitumor immunity have been widely studied, the potential clinical translation is still hampered by the substantial lack in proofs derived from human samples (Tang et al., 2020b). PAAD has occupied over 85% of pancreatic cancer, which represents the malignancy from exocrine pancreas (Ryan et al., 2014). Hence, we mainly focused on developing a scoring system that integrates the pyroptosis and ferroptosis profiles of PAAD to better stratify pancreatic cancer. In this study, the PAAD samples were initially stratified into 4 cell death subtypes based on the median expression levels of coexpressed pyroptosis- and ferroptosis-related genes. The patients in the pyroptosis subtype had relatively best survival, while the relatively worse survival was found in the mixed subtype. The TMB that reflects the mutation accumulation in cancer has shown negative correlation with the OS of patients, even when accounting for distinct cancer types (Pleasance et al., 2020). Across the 4 cell death subtypes, the patients in the ferroptosis and mixed subtypes presented higher TMB than that of patients in other subtypes. These findings together suggested the pyroptosis and ferroptosis might act synergistically to facilitate tumor progression in PAAD, even though patients with independently high expression of pyroptosis-related genes demonstrated the best outcome. Therefore, a comprehensive characterization of the distinct cell death processes would be a better approach to stratify PAAD for further individualized assessment and therapy.

Given the heterogeneity among different patients, it was more practical to quantify the distinct cell death processes in an individual tumor sample. In the present study, the P-F score was established by utilizing the Boruta algorithm. Patients with high P-F scores exhibited a longer survival time, suggesting that the P-F score might work as an indicator of favorable prognosis. The high predictive efficacy of the P-F score in PAAD for 1-, 2-, 3-, 4-, and 5-year survival times was confirmed by the analysis of ROCs. Through the univariate and multivariate Cox regression analyses, the P-F score was identified as an independent protective factor for OS. Meanwhile, good accuracy of the P-F score in predicting OS was further validated in the TCGA-PAAD, ICGC-PACA-CA, and E-MTAB-6134 cohorts *via* time-dependent AUCs. Our data collectively confirmed the well predictive ability of the P-F score, which could have a clinical application in assessing the OS of PAAD patients. As a key driving force for pancreatic tumorigenesis, *KRAS* mutations were found in nearly all PAAD (Waters and Der, 2018). Intriguingly, significantly elevated genomic mutations were noticed in the low P-F score group. For instance, the mutation frequencies of *KRAS* in the low and high P-F score groups were 92 and 62%, respectively. Recently, the activation of ferroptosis by high-iron diets or depletion of Gpx4 has been proven to promote *KRAS*-driven pancreatic tumorigenesis (Dai et al., 2020). Notably, nearly all the patients in the ferroptosis subtype were allocated in the low P-F score group. These findings suggested that *KRAS* mutations might drive the cell death profile toward a pattern of low P-F score in PAAD. Therefore, the P-F scoring system established in this study could effectively stratify PAAD in the fields of prognosis and genomic alterations.

As an emerging novel therapeutic choice, ICBs have displayed promising effects in multiple malignancies. Given the immune-privileged nature of PAAD, the majority of PAAD patients do not respond well to the ICBs. Accordingly, identifying the portion of patients who might benefit from the ICBs is an urgent demand for clinical practice. Through functional enrichment analysis, the genes that were involved in immunostimulating pathways, including T-cell activation and positive regulation of the immune effector process, were enriched in the high P-F score group. Additionally, a more activated immune phenotype with significantly higher intratumoral infiltrations of activated CD4^+^ memory T cells, CD8^+^ T cells, and γδ T cells was found in the high P-F score group, while the low P-F score group was associated with significantly higher infiltration of immunosuppressive Tregs. Emerging evidences have indicated that the preexisting anticancer immunity could positively regulate the response to immunotherapy, while the tumor-infiltrating Tregs could not only suppress the native anticancer immune response but also weaken the efficiencies of ICBs (Kim and Chen, 2016; Chen et al., 2019; Gonzalez-Navajas et al., 2021). The patients from the IMvigor210 cohort who received anti-PD-L1 treatment were evaluated. In line with these findings, a significantly higher response rate to anti-PD-L1 immunotherapy was observed in patients with high P-F scores. The high TMB has been routinely regarded as an indicator in predicting the response to ICB treatment (Cristescu et al., 2018; Chan et al., 2019). However, the high TMB was recently proven to fail in predicting response to treatment with ICBs across all solid cancer types (McGrail et al., 2021). Hence, the P-F score might be used as an alternative biomarker in predicting the response to ICB treatment. Overall, these results suggested that the patients with high P-F score might benefit more from the single agent of ICB treatment. Considering the enriched intratumoral Tregs in the low P-F score group, the depletion or suppression of Tregs might work in synergy with ICB treatment. In this content, there are ongoing clinical trials (ClinicalTrials.gov: NCT03447314, and NCT03739710) to validate the effects of the combination of ICBs and targeting Tregs in advanced solid tumors. These reasonable speculations in this study need future clinical trial-based validation in a large PAAD cohort.

Recently, some studies have explored the pyroptosis- or ferroptosis-related molecular subgrouping in various cancers, including PAAD, lung adenocarcinoma, and hepatocellular carcinoma (Tang et al., 2020a; Fu and Song, 2021; Liu et al., 2021). Some strengths could be noticed in our study. This study is more valuable and convincing when compared with other studies that only focused on pyroptosis- or ferroptosis-related genes (Tang et al., 2020a; Fu and Song, 2021; Liu et al., 2021). Different from one recent study that clustered the hepatocellular carcinoma based on the prognosis-related genes associated with ferroptosis and pyroptosis, this study stratified cell death subtypes based on dual analysis of pyroptosis- and ferroptosis-related genes and further established a scoring system depending on the DEGs among different cell death subtypes (Huo et al., 2021). The P-F scoring system established in our study could provide a better understanding of the crosstalk between the pyroptosis and ferroptosis in PAAD. Although an earlier study that focused more on the association between the immune populations and prognosis has provided an integrated immunophenotypic classification of PAAD, our study further broadened the genetic and immunologic variables to prognostic and therapeutic decision-making values (Wartenberg et al., 2018). Meanwhile, there are some limitations in this study. First, as limited by lacking therapeutic data of a PAAD-related immunotherapy cohort, we validated the availability of the P-F score in predicting the benefits from immunotherapy through utilizing the IMvigor210 cohort, an immunotherapy cohort of urothelial cancer. Besides, therapeutic data in the ICGC-PACA-CA, E-MTAB-6134, GSE57495, GSE21501, and GSE85916 datasets are incomplete. As a result, we just investigated the effect of the P-F score in the patients who have received chemotherapy in the TCGA-PAAD cohort. Notwithstanding its limitations, our study does establish an integrated P-F scoring system in PAAD. Large-scale clinical trials are required to further verify the value of the P-F score in predicting the benefits from immunotherapy and chemotherapy for PAAD.

In conclusion, we comprehensively analyzed the landscape of pyroptosis and ferroptosis in PAAD, establishing an integrated P-F scoring system. Distinct genomic alterations, immune infiltrations, and survival were revealed between the high and low P-F score groups. Functionally, the P-F score has superior capacity in predicting the outcomes and responses to the treatments with chemotherapeutic agents or immunotherapies of PAAD patients. Collectively, the systematic evaluation of the tumor cell death profiles conducted by this study has potential values for prognostic evaluation and therapeutic decision-making for PAAD patients.

## Data Availability

Publicly available datasets were analyzed in this study. These data can be found here: The RNA-sequence (RNA-Seq) data with matched clinical information of all available PAAD patients were extracted from The Cancer Genome Atlas (TCGA) (https://www.cancer.gov/tcga) (n = 176) and International Cancer Genome Consortium (ICGC) (https://daco.icgc.org/) (n = 165) databases. Additionally, the E-MTAB-6134 dataset with completely clinical information of 288 PAAD patients and datasets without detailed clinical information including GSE57495, GSE21501, and GSE85916 were all extracted from the Array Express database (https://www.ebi.ac.uk/arrayexpress). The TCGA dataset was utilized as the training cohort, and the other datasets were set as validation cohorts. Based on the Creative Commons 3.0 license downloaded from http://research-pub.gene.com/IMvigor210CoreBiologie, the IMvigor210 dataset was extracted from a freely available data package. The IMvigor210 dataset containing 298 patients of urothelial cancer who had received immunotherapy was performed to validate the prediction value of the P-F score. The corresponding information of somatic mutations in TCGA-PAAD patients was extracted from UCSC Xena (https://xena.ucsc.edu/).
