# Supplementary figures and images for: Integratively Genomic Analysis Reveals the Prognostic and Immunological Characteristics of Pyroptosis and Ferroptosis in Pancreatic Cancer for Precision Immunotherapy
Source: Front Cell Dev Biol. 2022 Feb 15;10:826879. doi: 10.3389/fcell.2022.826879 (PMC8885993; doi:10.3389/fcell.2022.826879)

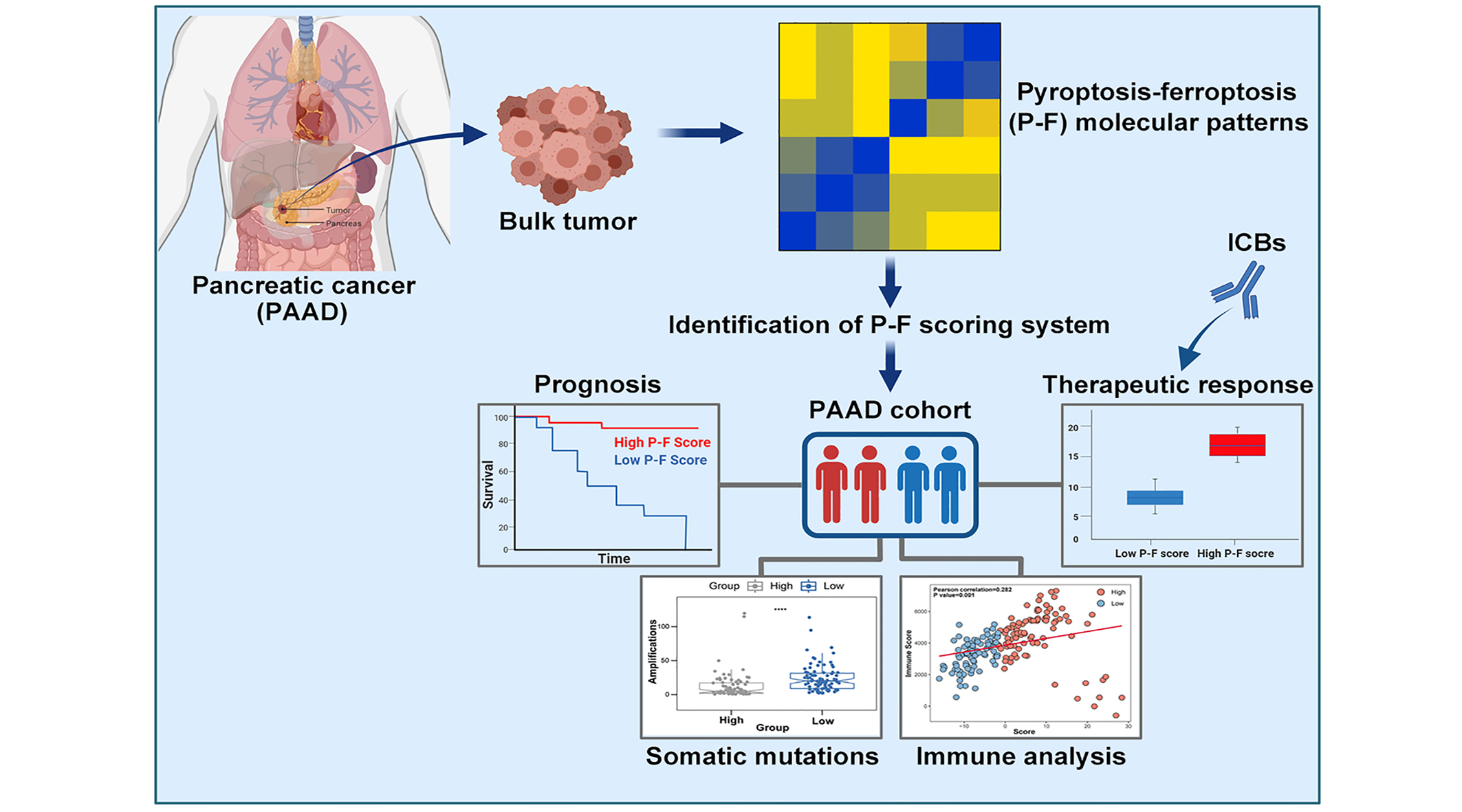

Supplement: Supplementary file 4 [file Image1.TIF]
