# Supplementary material for: Integratively Genomic Analysis Reveals the Prognostic and Immunological Characteristics of Pyroptosis and Ferroptosis in Pancreatic Cancer for Precision Immunotherapy
Source: Front Cell Dev Biol. 2022 Feb 15;10:826879. doi: 10.3389/fcell.2022.826879 (PMC8885993; doi:10.3389/fcell.2022.826879)
Supplement: Supplementary file 6 [file DataSheet1.PDF]

Supplementary Material

Supplementary Figures

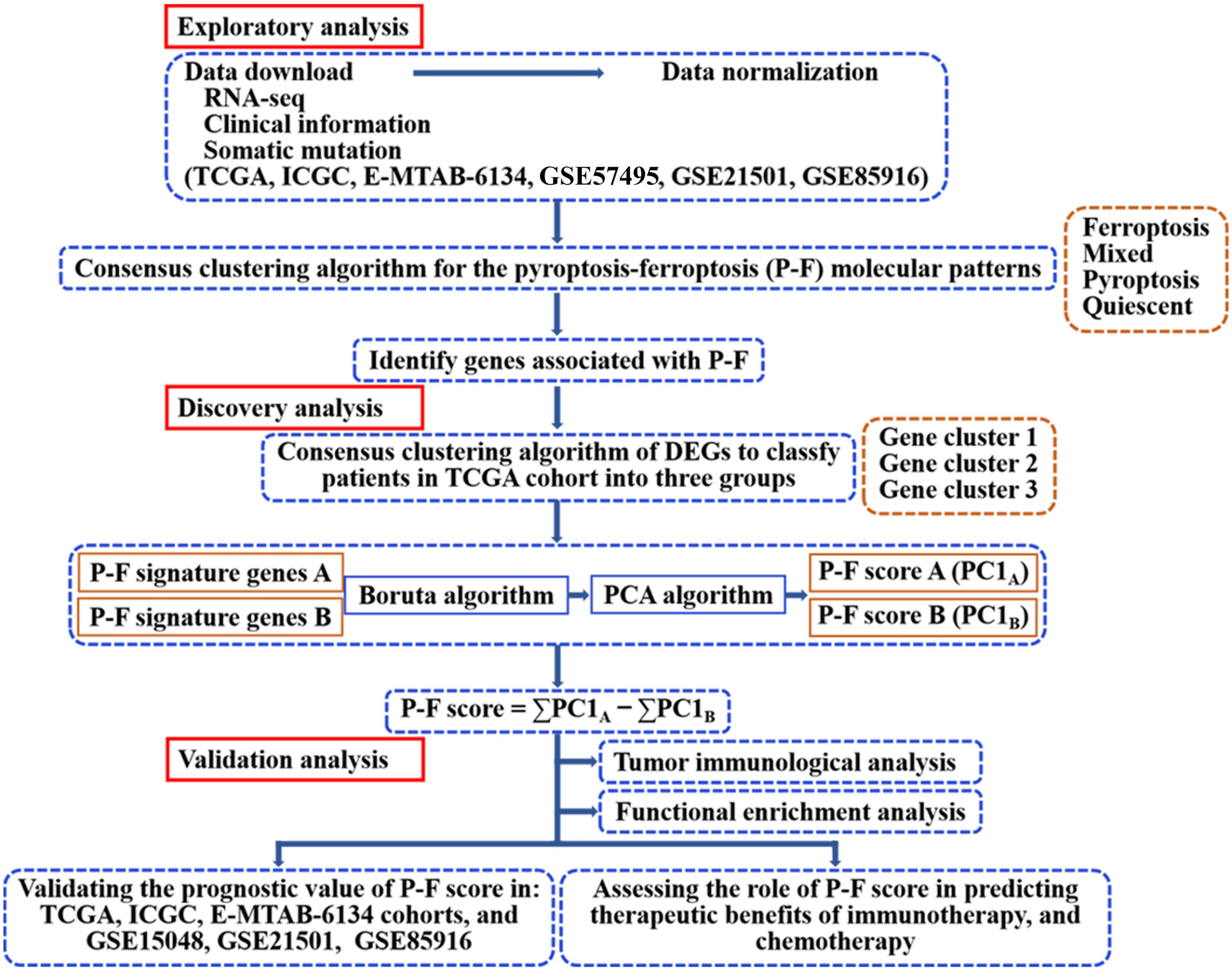

Supplementary Figure 1. The flow diagram of P-F score development.

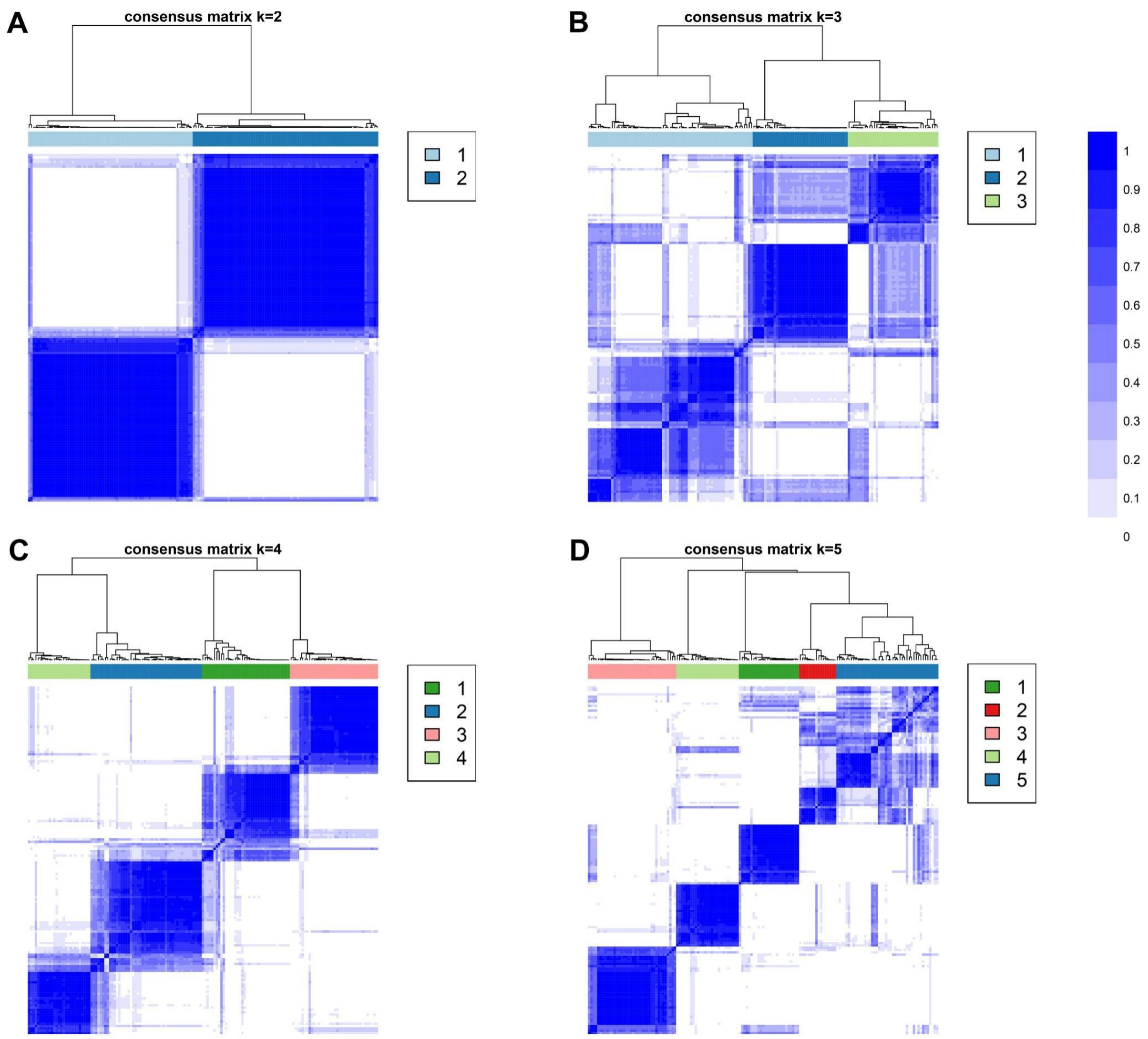

Supplementary Figure 2. Consensus matrixes of PAAD samples for each k (k = 2-5).

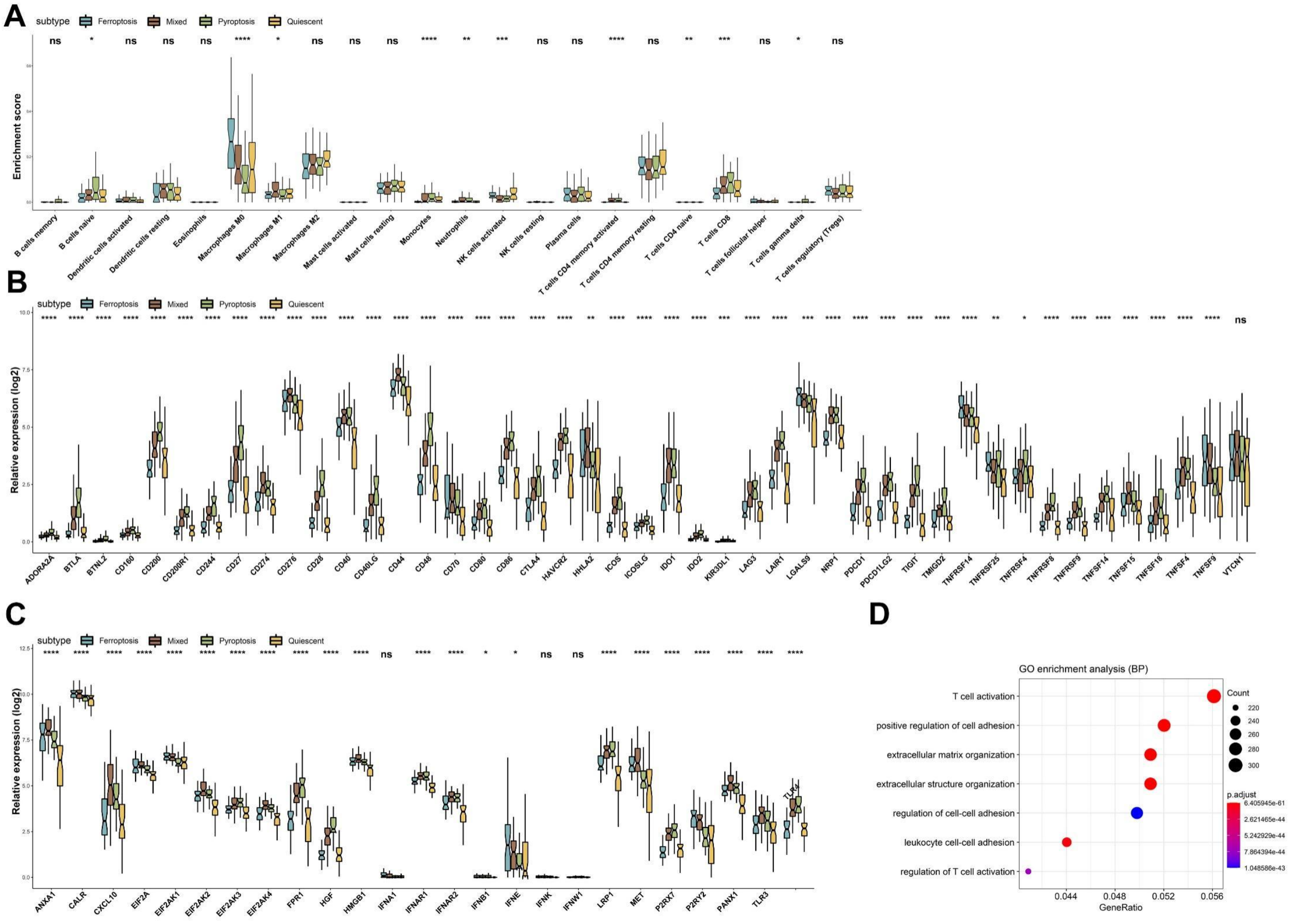

Supplementary Figure 3. Tumor immunological analyses across the four cell death subtypes and GO enrichment analysis of the DEGs. Comparisons about the abundances of 19 immune infiltrations **(A)**, relative expressions of the ICPs-relevant genes **(B)** and ICDs-relevant genes **(C)** across the four subtypes. **(D)** GO enrichment analysis of the DEGs that enriched in biological processes.

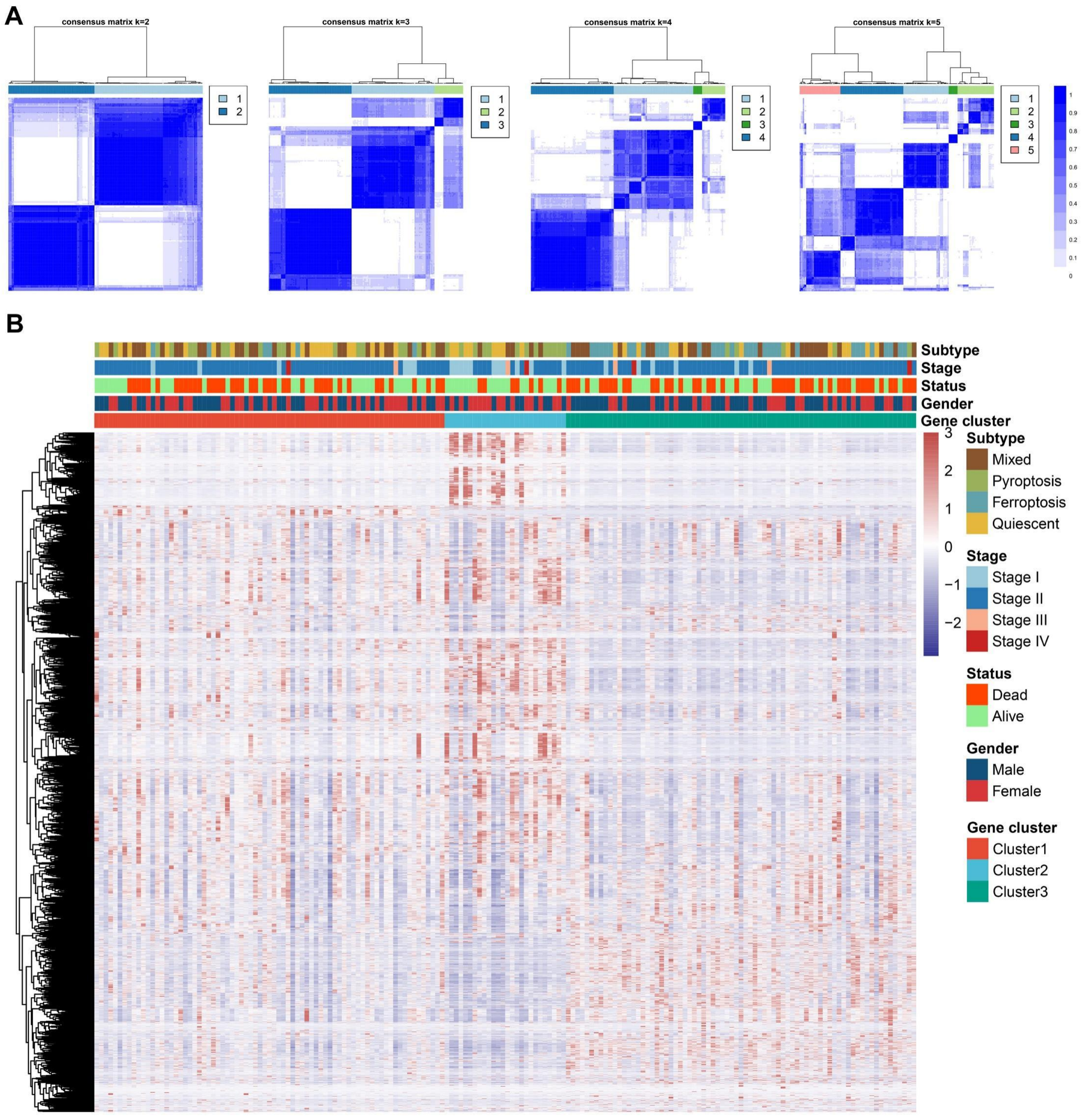

Supplementary Figure 4. Consensus clustering of the DEGs among the four cell death subtypes. **(A)** Consensus matrixes of TCGA-PAAD cohort for each k (k = 2-5). **(B)** Unsupervised clustering of the DEGs among the four cell death subtypes to further stratify PAAD patients into three gene clusters.

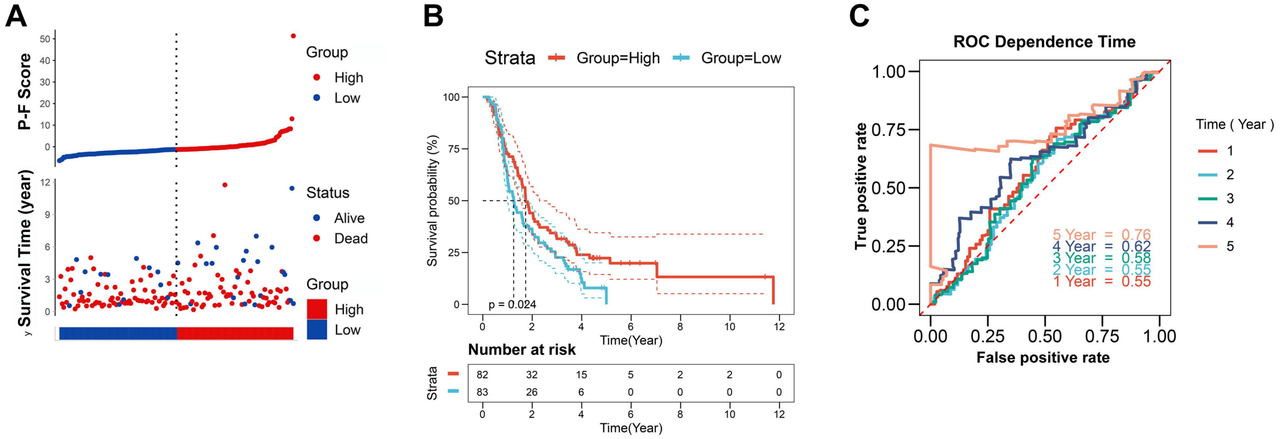

Supplementary Figure 5. Validation of the P-F score in ICGC-PACA-CA cohort. **(A)** Distribution of the P-F score and survival status of PAAD patients in ICGC-PACA-CA cohort. **(B)** Survival analysis of the PAAD patients in high and low P-F score groups in ICGC-PACA-CA cohort (log-rank *P* = 0.024). **(C)** ROC curves for the 1-, 2-, 3-, 4-, and 5-year survival times based on the P-F score in ICGC-PACA-CA cohort.

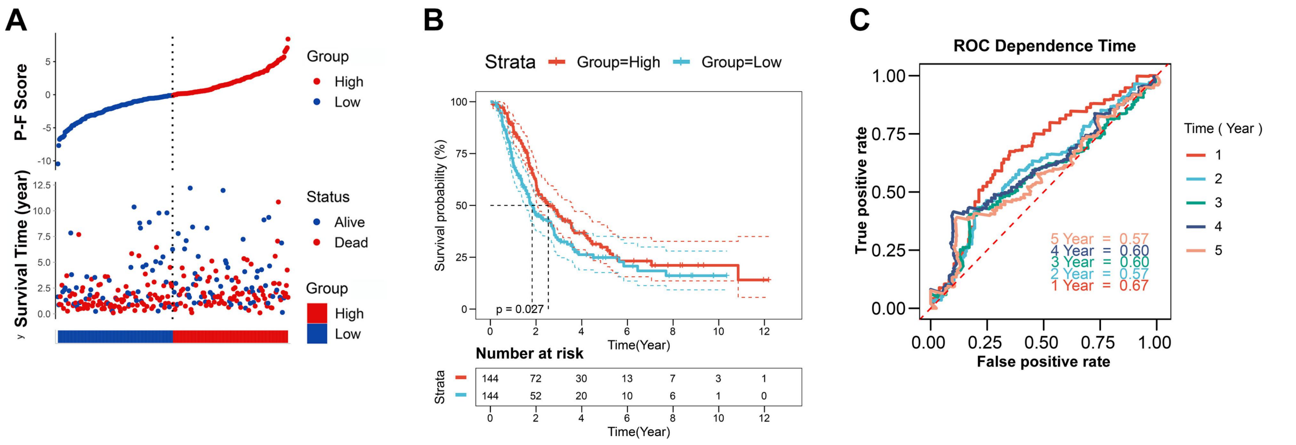

Supplementary Figure 6. Validation of the P-F score in E-MTAB-6134 dataset. **(A)** Distribution of the P-F score and survival status of PAAD patients in E-MTAB-6134 cohort. **(B)** Survival analysis of the PAAD patients in high and low P-F score groups in E-MTAB-6134 cohort (log-rank *P* = 0.027). **(C)** ROC curves for the 1-, 2-, 3-, 4-, and 5-year survival times based on the P-F score in E-MTAB-6134 cohort.

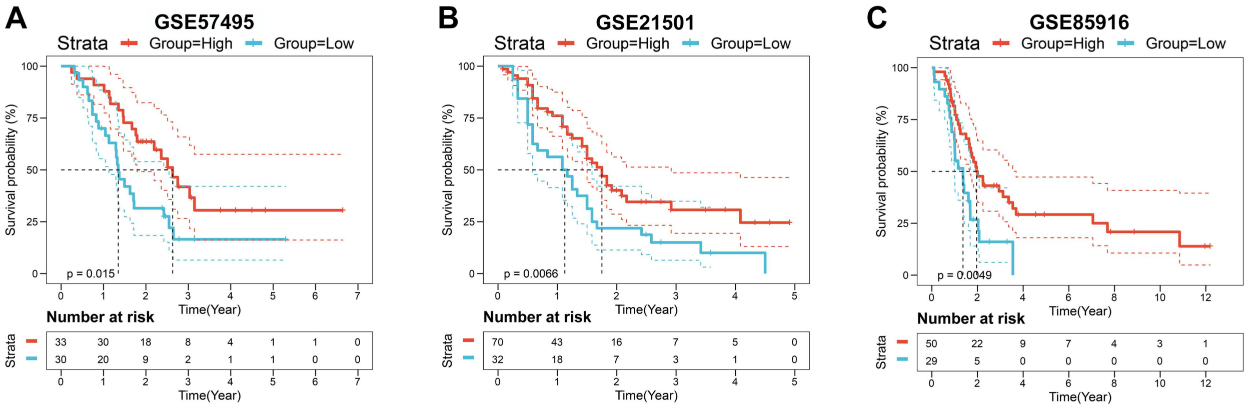

Supplementary Figure 7. Kaplan-Meier curves for the patients with high or low P-F scores in GSE57495, GSE21501, and GSE85916.

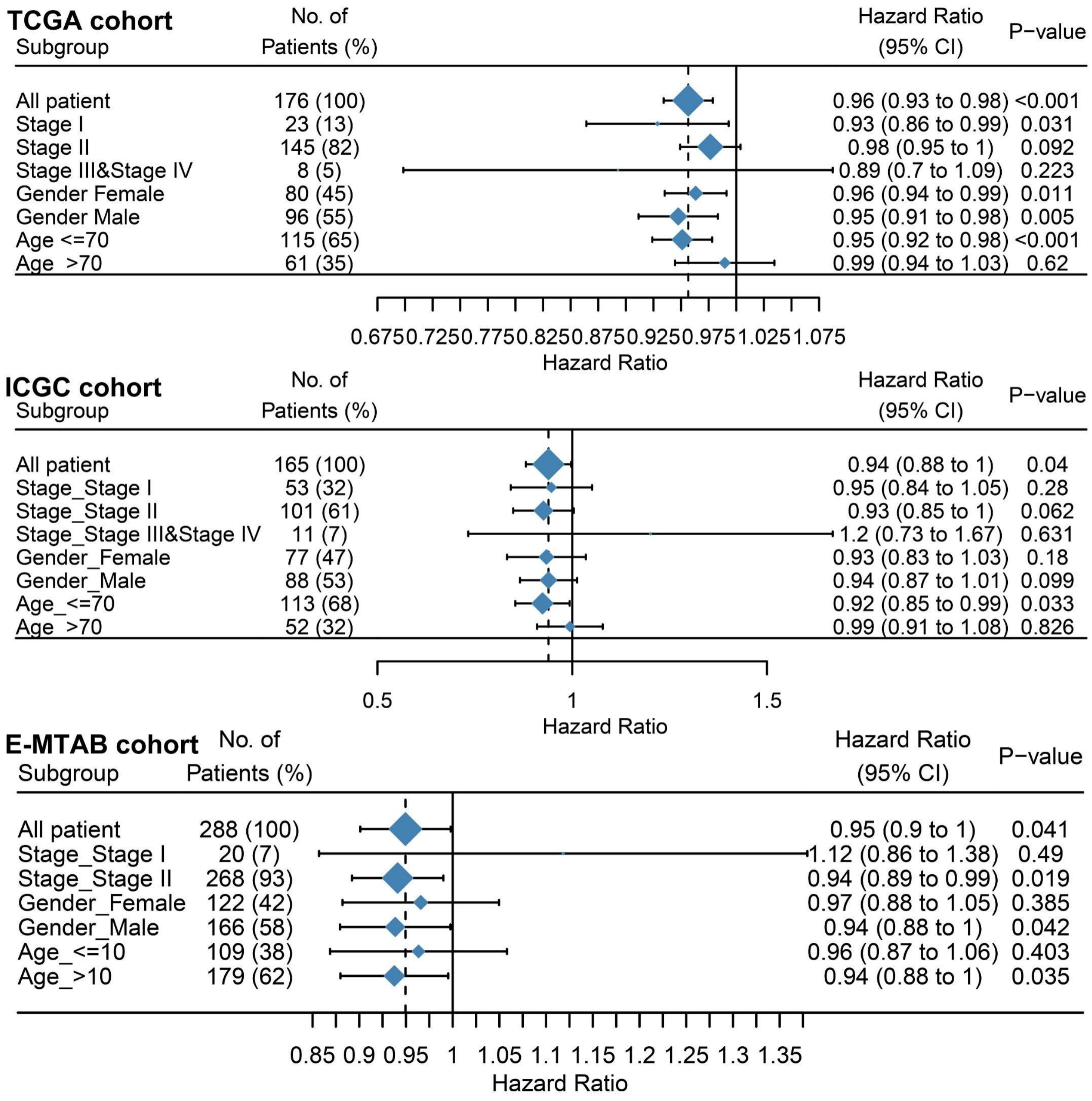

Supplementary Figure 8. Stratified analysis for the high and low P-F score groups. This analysis was performed in PAAD patients with different stage, gender, and age.

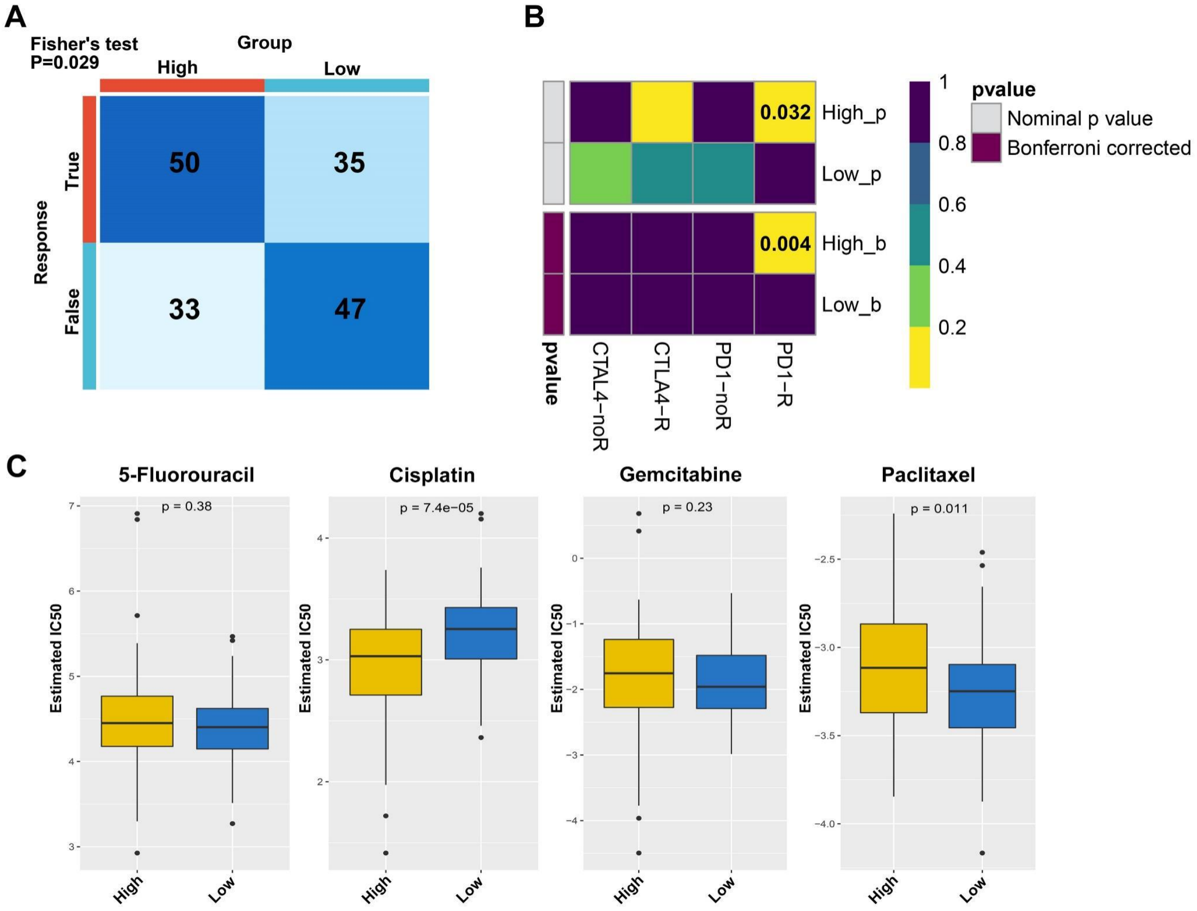

Supplementary Figure 9. The role of the P-F score in predicting the benefits from immunotherapy and chemotherapy in ICGC-PACA-CA cohort. **(A)** The responses to anti-PD-1 and anti-CTLA4 immunotherapies of PAAD patients with high or low P-F scores in ICGC-PACA-CA cohort were predicted by using the TIDE algorithm (Fisher's test *P* = 0.029). **(B)** Heatmap visualized the responses to anti-PD-1 and anti-CTLA4 immunotherapies between the high and low P-F score groups. **(C)** The estimated IC50 levels of 5-fluorouracil, cisplatin, gemcitabine, and paclitaxel between the two groups.

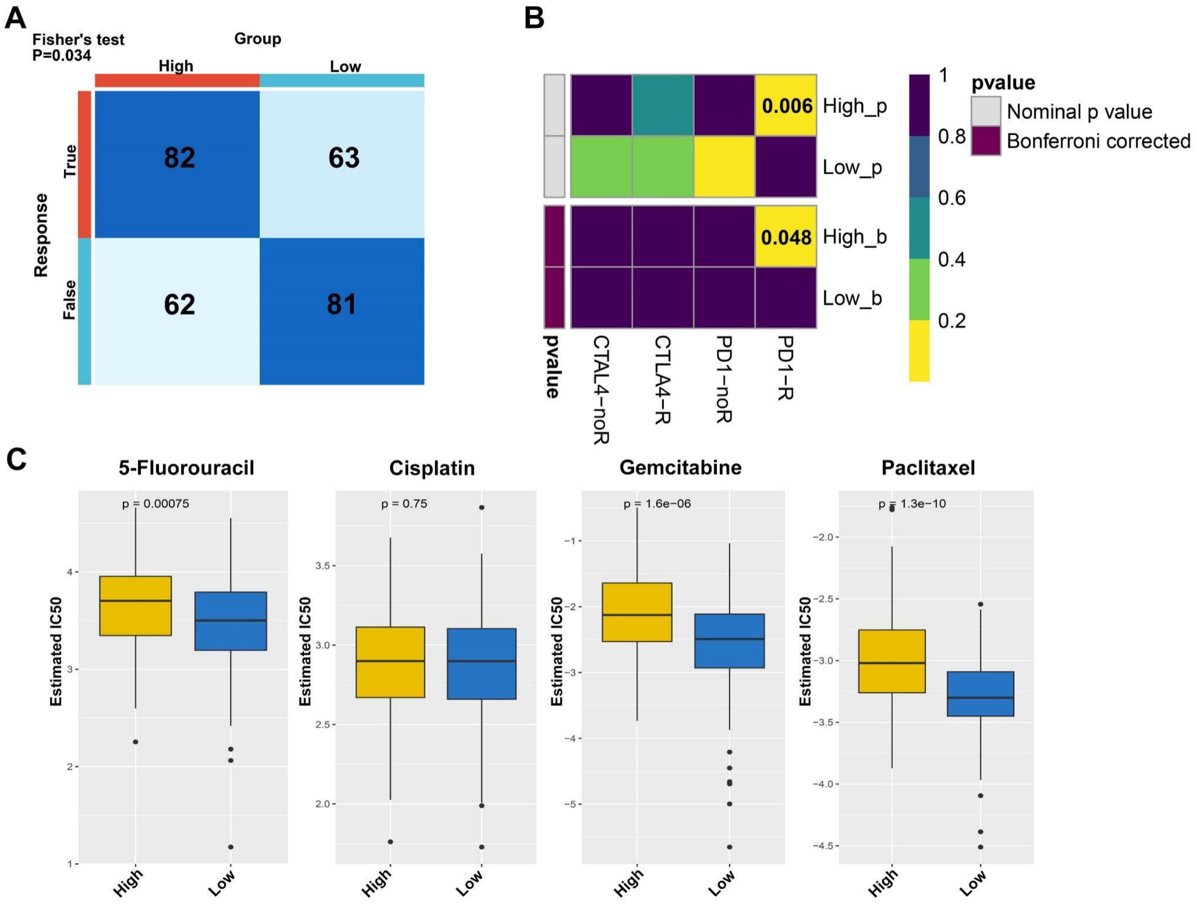

Supplementary Figure 10. The role of the P-F score in predicting the benefits from immunotherapy and chemotherapy in E-MTAB-6134 cohort. **(A)** The responses to anti-PD-1 and anti-CTLA4 immunotherapies of PAAD patients with high or low P-F scores in E-MTAB-6134 cohort were predicted by using the TIDE algorithm (Fisher's test *P* = 0.034). **(B)** Heatmap visualized the responses to anti-PD-1 and anti-CTLA4 immunotherapies between the high and low P-F score groups. **(C)** The estimated IC50 levels of 5-fluorouracil, cisplatin, gemcitabine, and paclitaxel between the two groups.

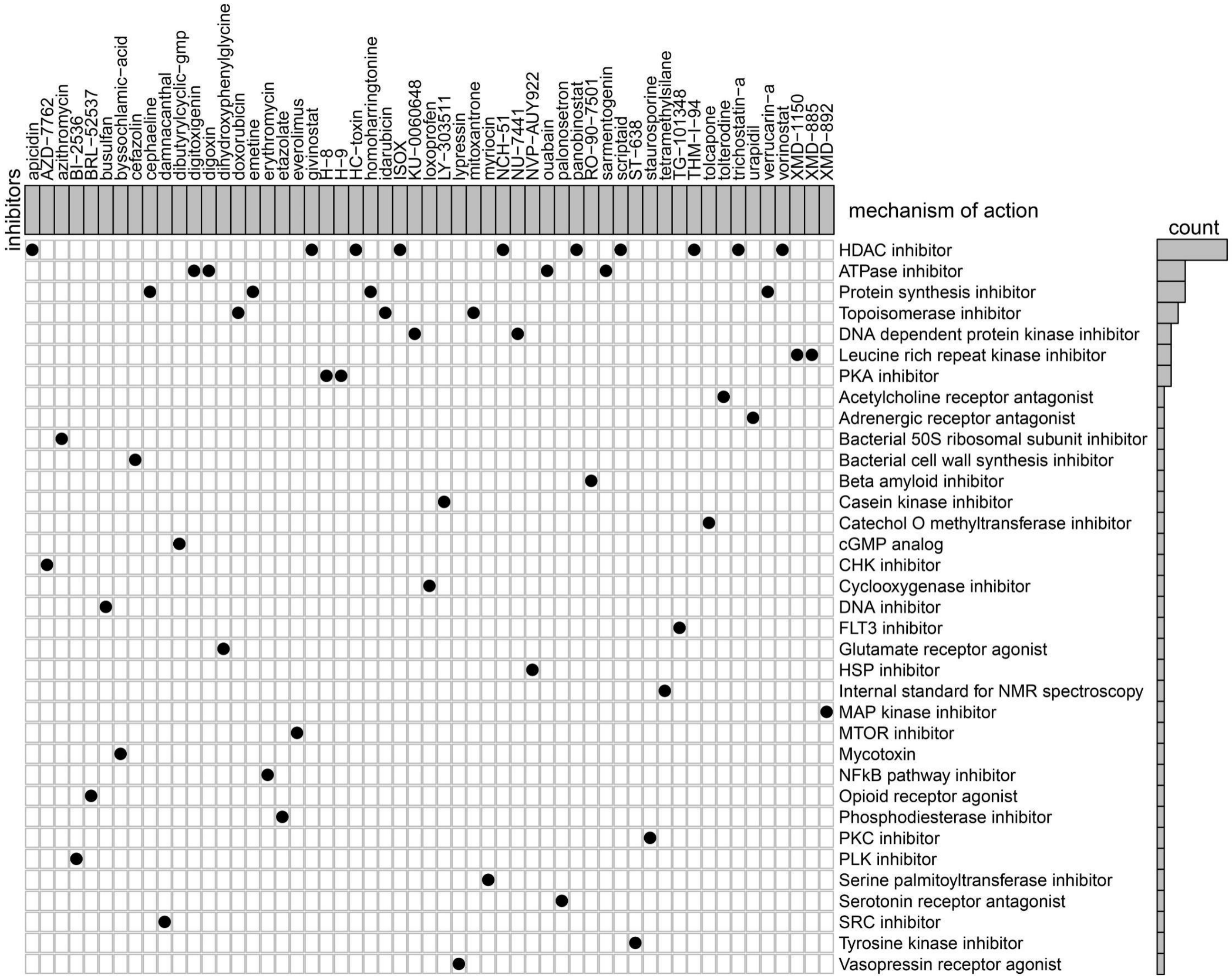

Supplementary Figure 11. Heatmap depicting the small molecular compounds (perturbagen) in column and their shared mechanism of actions in rows by using the CMap database.

Supplementary Tables

Table S1. The gene sets of pyroptosis.

Table S2. The gene sets of ferroptosis.

Table S3. The 6164 DEGs identified across four cell death subtypes.

Table S4. GO functional enrichment of DEGs across four cell death subtypes.

Table S5. The DEGs that referred to the corresponding P-F gene signatures.

Table S6. The 133 most abundant DEGs that identified among the genomic clusters.

Table S7. The GO functional enrichment of up-regulated DEGs in high P-F score group.

Table S8. The GO functional enrichment of down-regulated DEGs in high P-F score group.

Table S9. The GSEA analysis of the pathways enriched in high P-F score group.

Table S10. The GSEA analysis of the pathways enriched in low P-F score group.

Table S11. The MoA analysis revealed the underlying mechanism of drug actions.
